# Breaking the waves: improved detection of copy number variation from microarray-based comparative genomic hybridization

**DOI:** 10.1186/gb-2007-8-10-r228

**Published:** 2007-10-25

**Authors:** John C Marioni, Natalie P Thorne, Armand Valsesia, Tomas Fitzgerald, Richard Redon, Heike Fiegler, T Daniel Andrews, Barbara E Stranger, Andrew G Lynch, Emmanouil T Dermitzakis, Nigel P Carter, Simon Tavaré, Matthew E Hurles

**Affiliations:** 1 Computational Biology Group, Department of Applied Mathematics and Theoretical Physics, University of Cambridge, Centre for Mathematical Sciences, Wilberforce Road, Cambridge CB3 0WA, UK; 2 Computational Biology Group, Department of Oncology, University of Cambridge, Cancer Research UK Cambridge Research Institute, Robinson Way, Cambridge CB2 0RE, UK; 3 The Wellcome Trust Sanger Institute, The Wellcome Trust Genome Campus, Hinxton, Cambridge CB10 1SA, UK

## Abstract

**Background:**

Large-scale high throughput studies using microarray technology have established that copy number variation (CNV) throughout the genome is more frequent than previously thought. Such variation is known to play an important role in the presence and development of phenotypes such as HIV-1 infection and Alzheimer's disease. However, methods for analyzing the complex data produced and identifying regions of CNV are still being refined.

**Results:**

We describe the presence of a genome-wide technical artifact, spatial autocorrelation or 'wave', which occurs in a large dataset used to determine the location of CNV across the genome. By removing this artifact we are able to obtain both a more biologically meaningful clustering of the data and an increase in the number of CNVs identified by current calling methods without a major increase in the number of false positives detected. Moreover, removing this artifact is critical for the development of a novel model-based CNV calling algorithm - CNVmix - that uses cross-sample information to identify regions of the genome where CNVs occur. For regions of CNV that are identified by both CNVmix and current methods, we demonstrate that CNVmix is better able to categorize samples into groups that represent copy number gains or losses.

**Conclusion:**

Removing artifactual 'waves' (which appear to be a general feature of array comparative genomic hybridization (aCGH) datasets) and using cross-sample information when identifying CNVs enables more biological information to be extracted from aCGH experiments designed to investigate copy number variation in normal individuals.

## Abstract 2

Datasets used for detecting copy number variation (CNV) are shown to be affected by a technical artifact. A novel CNV calling algorithm is presented which removes this artifact and identifies regions of CNV better than existing methods.

## Background

Copy number variation (CNV) throughout the human genome has recently been the focus of much interest and array comparative genomic hybridization (aCGH) technology has been instrumental in identifying regions of the genome where CNVs occur. It is believed that such variation may explain the presence and development of adverse phenotypes ranging from HIV-1 infection to Alzheimer's and Parkinson's disease [1]. To analyze the experimental aCGH data and to identify the location of CNVs, specific statistical tools for normalization and CNV calling (segmentation) are undergoing continual refinement and development.

There are many algorithms [2,3] for segmenting aCGH data into classes with differing numbers of copies. The vast majority of these algorithms identify CNVs by identifying outlier regions within a single genome (cross-genome analysis), and do not increase statistical power by examining the same genomic region across many samples (cross-sample analysis). This is partly due to the fact that many of these algorithms have been designed for application to tumor data, where rearrangements are frequently very large (covering many megabases), the locations of breakpoints differ between samples [2] and sample heterogeneity prohibits accurate estimation of copy number. Recently, there has been dramatic growth in the size and number of aCGH datasets examining constitutive CNVs, where rearrangements are smaller (and thus harder to identify through cross-genome analysis alone) and breakpoints are shared due to common ancestry [4]. This motivates the development of improved CNV calling algorithms that incorporate both cross-sample and cross-genome information and can separate samples into groups that represent genuine differences in copy number. Such approaches are by their very nature more likely to be sensitive to technical artifacts that result in systematic differences between samples, and thus require the development of improved normalization procedures to minimize these effects. One of the few examples where both cross-sample and cross-genome information has been utilized is in [5] where the authors developed a pseudo-likelihood based method that determined the location of breakpoints and allocated clones into groups that can be classified as gain, loss or normal. However, in general, where cross-sample information has been used, it has focused on combining segmentation patterns across a cohort of patients [6-8].

Recently, whole genome tiling path (WGTP) arrays covering 93.7% of the genome with large-insert clones have been used to find CNVs in a large collection of apparently healthy individuals [1,9]. In [1], the CNVfinder algorithm [9] was used to identify clones that correspond to CNVs. CNVfinder is a threshold-based method and the thresholds are set conservatively to reduce the number of false positive calls. In this paper, we describe a mixture-model based method, CNVmix, for identifying CNVs that takes advantage of cross-sample information. The development of CNVmix was impeded by the presence of a significant technical artifact (spatial autocorrelation, or 'wave') observed in the data used in [1]. We describe how the wave effect can be modeled and removed, leading to an improved clustering of the data and better calling of CNVs by CNVfinder. Subsequently, we show that CNVmix can be applied to the wave corrected data in order to separate samples into groups that represent genuine differences in copy number, leading to more CNVs being detected in each experiment.

## Results and discussion

### Spatial autocorrelation in aCGH data

**Figure 1:**
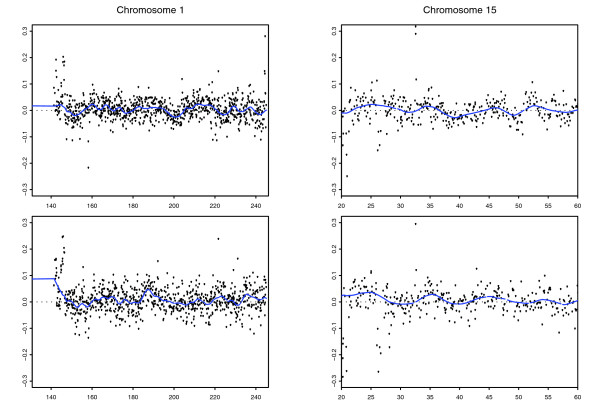
Examples of the wave artifact and the fitted loess curve. In the two left hand plots we display the log_2 _ratios for clones on the long arm of chromosome 1 plotted against their genomic location for two HapMap samples, NA06993 (top) and NA11832 (bottom). On the right we plot the log_2 _ratios for clones on a section of the long arm of chromosome 15 against their genomic location for samples NA06993 (top) and NA11832 (bottom). The wave effect can be observed by scanning across each of the plots from left to right. The fitted loess curves for each of these samples/genomic regions have been overlaid in blue.

The HapMap collection [10] consists of 270 samples split between four populations: 30 parent-offspring trios of European descent from Utah (CEU), 30 parent-offspring trios of Yorubans from Nigeria (YRI), 45 Japanese from Tokyo (JPT) and 45 Han Chinese from Beijing (CHB). In [1], cell-line DNA samples taken from 269 members of this collection were hybridized to WGTP arrays along with the remaining HapMap sample (an offspring from the CEU population) used as a common reference. The data were normalized as described in [1] and (the raw and normalized data) can be downloaded from [11]. On examination of genome-wide and chromosome-specific plots of the normalized data, we observed a spatially autocorrelated 'wave' pattern (Figure 1). This wave pattern was consistent across samples in that the peaks and troughs aligned well (Figure 1). However, the amplitude of the wave varied from sample to sample and, in a small number of samples, the direction of the wave was reversed. We deduced that this variation in amplitude would introduce noise that would mask our ability to obtain biologically meaningful clustering of individuals. Further, this variation in wave amplitude affects the clustering of log_2 _ratios at a given clone and, therefore, limits the ability to infer the relative diploid genome copy number of individuals [1].

**Figure 2:**
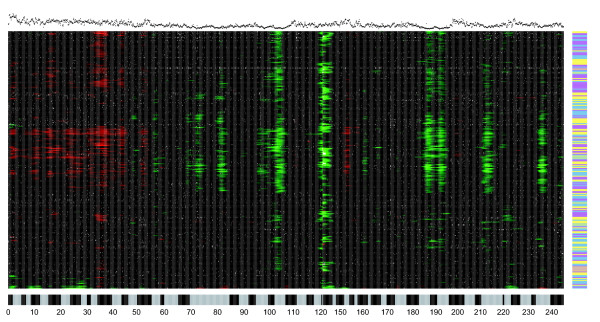
Ordered heatmap of fitted loess values. The clones on chromosome 1 are ordered along the x-axis and the HapMap samples not excluded because of the presence of chromosome-wide aberrations are plotted on the y-axis. A green/red region on the heatmap indicates that the fitted loess values in this region are consistently greater/less than zero. The samples have been ordered using the Ward agglomeration method and a Euclidean distance metric. The plot across the top of the heatmap indicates the GC content of each probe and the color bar on the right of the heatmap displays the ethnic origin of a sample: blue (YRI), yellow (CEU) and purple (CHB + JPT). The scale along the bottom gives the location of the cytobands on chromosome 1. The centromeric region is not covered by clones and has been excised from this plot, which creates the slight discontinuity at 120 Mb.

To quantify the wave effect and, more importantly, to explore how consistent it was across samples, we fitted a loess curve to the log_2 _ratios on each sample/chromosome independently whilst ensuring that the fit was not confounded by potentially real CNVs (see Materials and methods). Subsequently, we displayed the fitted loess curves using heatmaps. A heatmap for chromosome 1 is shown in Figure 2 and plots for the other chromosomes are contained in Additional data file 1. We observe from Figure 2 that the location of the wave is remarkably consistent across virtually all samples. There did not seem to be any major differences in the 'waviness' of data from different ethnic groups, as assessed by unsupervized clustering of the fitted loess values using the Ward agglomeration method (Figure 2) [12]. In addition, the periodicity of the wave is variable but is far longer than the typical size of a CNV. Given this difference in length between the waves and CNVs, by carefully modeling the wave it should be possible to reduce the amount of noise present in the data without removing biological variation. Finally, there is a strong correlation between the wave and the GC content of the clones - the Pearson correlation between the mean loess curve (averaged over all samples) and the GC content of the clones on chromosome 1 is -0.66. In other words, regions with a low GC content correspond roughly to peaks of the wave while regions with high GC content correspond to troughs. We determined that fitting a loess curve was preferable to correcting for GC content directly (linear regression), by showing that the former removes approximately twice as much noise (variance) from the data (Additional data file 2).

Wave artifacts have previously been reported in aCGH data [2] but have only been mentioned in passing. Consequently, little work has been done to determine the reasons for their existence. While Figure 2 suggests that the effect could be related to the GC content of each probe, this may not be the cause since GC content is highly correlated with many different genomic characteristics (gene density, repeat types, and so on) and can affect the efficiency of DNA labeling using dCTP-coupled fluorophores. We showed that simply replacing dCTP-coupled fluorophores with dUTP-coupled fluorophores did not remove, or invert the wave artifact (Additional data file 3). Determining the true cause of this spatial autocorrelation will require a detailed set of controlled experiments to distinguish between competing explanations. Consequently, it may be some time before we are able to ascertain experimentally the cause of the effect.

Motivated by the clear effect observed in Figure 2 and Additional data file 1, we explored whether normalizing the data by subtracting the wave effect: improved the clustering of the log_2 _ratios; improved the calling of CNVs using threshold-based approaches; and enabled the application of a novel CNV calling algorithm incorporating cross-sample information. Subsequently, we refer to the original (normalized) log_2 _ratios as 'uncorrected' and the wave-effect subtracted data as 'corrected'.

### The effect of the wave on genome-wide clustering

Prior work shows that it is possible to cluster the 210 unrelated HapMap samples into three groups that reflect their geographical ancestry (a CEU group, a YRI group and an East Asian (EAS) group composed of the JPT and CHB samples), using either single nucleotide polymorphism (SNP) or CNV genotypes [1,10]. We investigated whether we could recover this known population structure by clustering either the uncorrected or corrected WGTP data.

Before clustering, it is vital to account for artifacts that could add non-biological variability to the data and, thus, potentially confound the results. The most important artifact was related to a batch effect. The arrays were printed from two spotting sets, the main difference being the improved performance of clones on chromosome 15 in the later set. As a result there was systematic variability of chromosome 15 log_2 _ratios between these two sets of arrays. The second artifact affected the quality of the loess fit on chromosome 19. Due to difficulties in obtaining sequenced clones from inaccessible libraries, the coverage on this chromosome was only 68.4% compared to an average coverage of 93.7% for the entire genome. Since the accuracy of the loess fit depends upon the resolution of clones being high, it is difficult to estimate the curve accurately for this chromosome. Unsurprisingly, when we included data from these two chromosomes in the clustering, the resulting clusters did not reflect well the ethnic origin of the samples (Additional data file 4). This was the case for both the corrected and uncorrected data.

**Figure 3:**
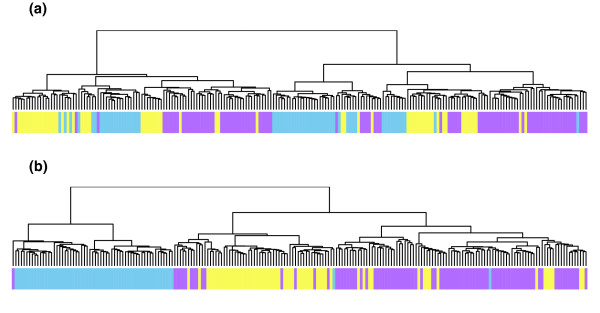
Dendrograms of the uncorrected and corrected log_2 _ratios. **(a) **Clustering of the uncorrected log_2 _ratios of the unrelated samples for all 22 autosomal chromosomes (excluding chromosomes 15 and 19). The heatbar under the dendrogram indicates the ethnic origin of the sample (blue, YRI; yellow, CEU; purple, CHB + JPT). **(b) **Clustering of the wave corrected log_2 _ratios of the unrelated samples on the same autosomal chromosomes.

After data from chromosomes 15 and 19 were excluded, we observed that clustering the uncorrected data (Figure 3a) still resulted in clusters that did not particularly well reflect the ethnicity of the samples. However, after clustering the corrected data (Figure 3b) we observed three major clusters - the LH cluster contains over 95% of the YRI samples, the far right cluster contains over 85% of the CHB and JPT samples and the middle cluster contains nearly 75% of the CEU samples. This pattern of clustering is consistent with that obtained from CNV genotypes in which the YRI exhibit the most distinct clustering [1]. This suggests that correcting for the wave effect can significantly aid the recovery of important biological information. It is also worth noting that clustering over categorized data may improve the separation of the populations still further. However, this would necessitate the application of a novel (in this context) distance metric and, consequently, is likely to be an interesting topic for future research.

### The effect of the wave on calling CNVs using threshold-based methods

As noted in the Background, one of the principal problems when using any threshold-based method to identify CNVs is that the thresholds have to be set conservatively to reduce the number of false positives. This can result in a failure to identify true CNVs. We suggest that one reason why thresholds have to be set conservatively is the wave effect. More importantly, if the thresholds are set too liberally, clones at the peak or trough of the wave may be incorrectly identified as CNVs.

One approach that could result in an increased number of CNVs being identified without a commensurate increase in the number of false positives would be to apply a threshold-based CNV calling method to the wave corrected data. We tested this hypothesis by applying the CNVfinder algorithm using the threshold multipliers that had been optimized for the uncorrected data. These thresholds are set on a chromosome/sample specific basis as multiples of the estimated standard deviation for that chromosome/sample [9]. Moreover, the algorithm incorporates different multipliers for detecting singleton CNVs and for finding runs of clones that represent a CNV [9]. Additional post-processing steps within the algorithm estimate the likely bounds of the CNV. Given the important role played by the signal-to-noise (S:N) ratio in the implementation of CNVfinder, we expected there to be an increase in the number of clones identified as CNVs after wave correction. To ensure that this did not coincide with a major increase in the number of false positive calls, we first examined the calls made by CNVfinder on the validated data described in [9].

Here, a well-studied diploid cell line (NA15510 - not a HapMap cell line) was hybridized to the common reference sample in five replicate experiments (labeled from A to E in order of increasing variability) and the copy number status of 137 clones was independently verified using quantitative multiplex PCR of short fluorescent fragments (QMPCR) or SYBR green real-time PCR [9]. (Validation was attempted for 154 putative CNV clones but 17 of these fell within rearranged intergenic regions or showed differential male/female response and, as they may not represent true CNVs, were excluded here.) Of the 137 clones, 112 were found to represent genuine CNVs while the remaining 25 were not. Subsequently, we used the sensitivity, specificity and the false discovery rate (FDR) to assess the performance of CNVfinder before and after wave correction. We note that the validated clones were not selected at random [9] but were chosen to be enriched for CNV (if 137 clones had been chosen at random, only 1 or 2 would have been expected to contain detectable CNVs in a given aCGH experiment, which would have given negligible power to estimate the FDR and sensitivity). As a consequence of this non-random sampling, the estimate of the specificity is likely to be underestimated and not truly representative of the population value. However, it is important to emphasize that all of the measures are valid for comparing the relative quality of different sets of calls within this study.

Upon examining the FDR of the CNVfinder algorithm before and after wave correction (Tables 1 and 2), we can observe that it is decreased slightly in two replicates (A and B) and increased in three replicates (C, D and E). However, this increased FDR in the noisier replicates is (unsurprisingly) accompanied by an increase in sensitivity without a dramatic loss of specificity (Tables 1 and 2). This illustrates that removing the wave can lead to an increase in the number of calls without a major increase in the number of false positive calls made, and gave us confidence that applying CNVfinder to the wave-corrected data could result in the identification of more clones that were likely to harbor CNVs.

**Table 1:** Performance of the CNVfinder algorithm using validated data before wave correction

		Calls				
		
Status	Number of regions	A	B	C	D	E
Unvalidated	25	4	4	3	0	0
Validated	112	68	70	65	47	35
Total	137	72	74	68	47	35
						
False discovery rate		6	5	4	0	0
Sensitivity (confidence interval)		61 (51, 70)	62 (53, 72)	58 (48, 67)	42 (33, 52)	31 (23, 41)
Specificity (confidence interval)		84 (66, 95)	84 (66, 95)	88 (71, 97)	100 (90, 100)	100 (90, 100)

The number of regions called in five replicate experiments (A to E, ranked by standard deviation) using CNVfinder before wave correction. The false discovery rate, sensitivity and specificity are quoted as percentages and the confidence intervals quoted are the 95% intervals.

**Table 2:** Performance of the CNVfinder algorithm using validated data after wave correction

		Calls				
		
Status	Number of regions	A	B	C	D	E
Unvalidated	25	4	3	4	3	2
Validated	112	71	70	75	57	36
Total	137	75	73	79	60	38
						
False discovery rate		5	4	5	5	5
Sensitivity (confidence interval)		63 (54, 72)	62 (53, 72)	67 (57, 76)	51 (41, 61)	32 (24, 42)
Specificity (confidence interval)		84 (66, 95)	88 (71, 97)	84 (66, 95)	88 (71, 97)	92 (77, 99)

The number of regions called in five replicate experiments (A to E, ranked by standard deviation) using CNVfinder after wave correction. The false discovery rate, sensitivity and specificity are quoted as percentages and the confidence intervals quoted are the 95% intervals.

Indeed, upon doing this, 596 additional (putative) CNV-harboring clones were identified. After post-processing was applied to find copy number variable regions (CNVRs) across all samples [1], virtually all regions (99.4%) identified by CNVfinder before wave correction were also flagged when CNVfinder was applied to the corrected data, which confirms that, first, the loess normalization does not remove known CNVs, and second, there is minimal evidence of wave-induced false positive CNV calls in [1].

The quality of the CNVfinder calls on the corrected data was further assessed by examining the frequency with which a clone was called as a CNV among the 270 HapMap individuals. It has previously been shown that observing a CNV in several individuals is a good indicator of a true positive CNV call [1]. We observed that the call frequency of CNVs was increased when CNVfinder was applied to the corrected data relative to the calls made from uncorrected data. Of the 837 regions commonly identified by CNVfinder before and after correction, 418 were called in more samples and only 7 were called in fewer samples. Moreover, 80 CNVRs previously identified in only a single sample were called in at least two samples.

**Figure 4:**
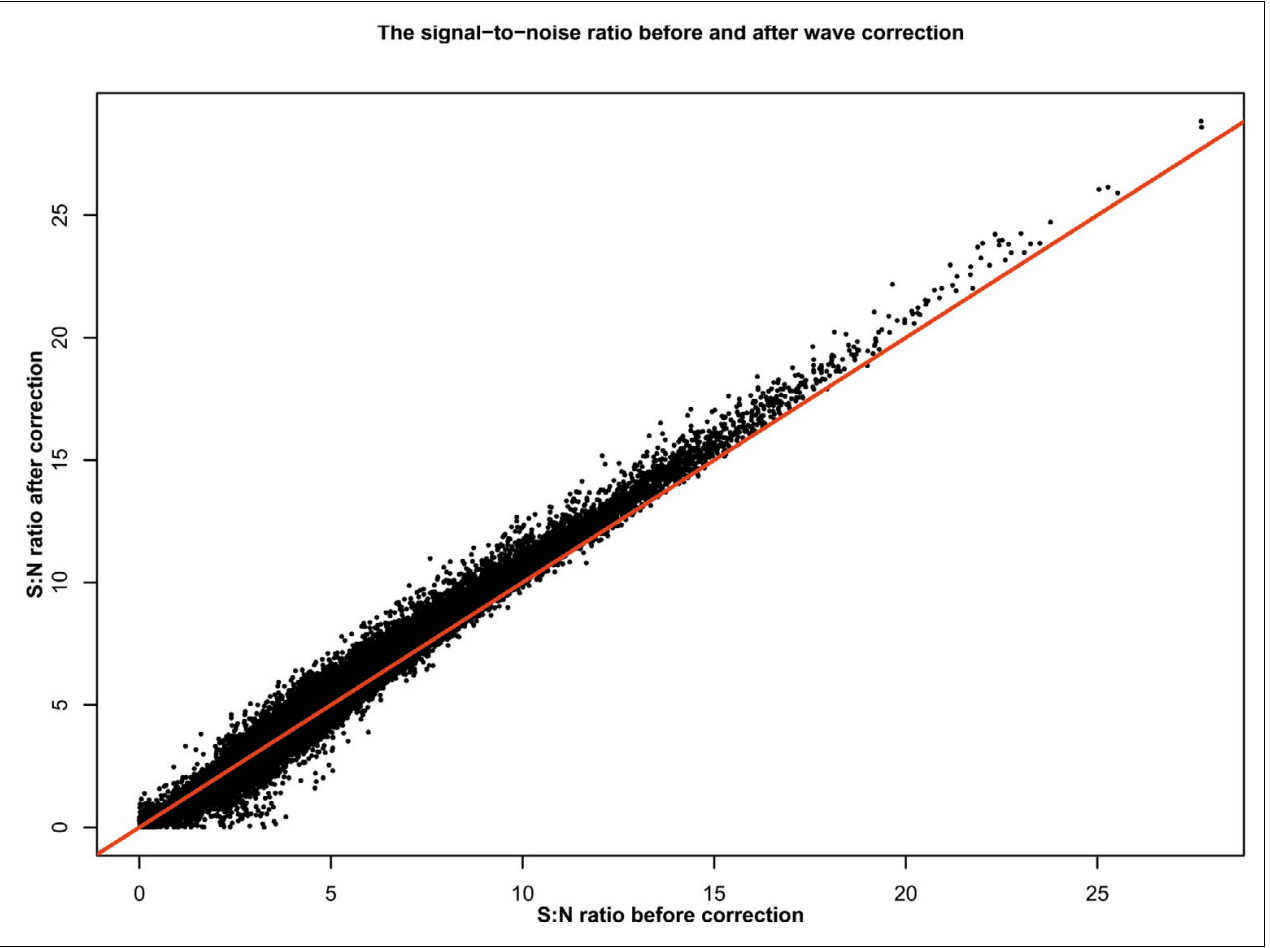
Comparing the S:N ratio before and after wave correction. The S:N ratios for all clones/samples called as CNVs in [1] calculated before and after wave correction are plotted on the x- and y-axes, respectively. For a clone flagged as a CNV in a given sample, the S:N ratio is defined as its log_2 _ratio divided by the standard deviation of all log_2 _ratios on the chromosome on which the CNV is located (for that sample). The red line has a slope of 1 and intercept 0. Points plotted above this line show an increase in S:N ratio after wave correction.

Finally, we compared the S:N ratio for all clones identified previously as putative CNVs [1] in uncorrected and corrected data. We observed (Figure 4) that, on average, the S:N ratio was significantly increased for the putative CNVs in the corrected data (*p *value < 10^-10^, median increase = 0.15 (3.5%), sign-test, null hypothesis: median increase = 0), confirming that the majority of these CNV-containing clones are more easily distinguished from the rest of the data after correcting for the wave effect.

### Model-based cross-sample CNV calling: CNVmix

Despite the improvement in the S:N ratio when the wave is corrected and the subsequent increase in the number of CNV harboring clones identified, threshold-based methods are limited in their ability to identify which samples have a CNV at a particular clone. For example, CNVfinder uses the same multipliers for all chromosomes/samples and to ensure that a small number of false positives are identified for the less variable samples, these multipliers are set conservatively (Tables 1 and 2). Consequently, far fewer calls are made for samples/chromosomes with higher variability, leading to an increase in the number of false negative calls made.

**Figure 5:**
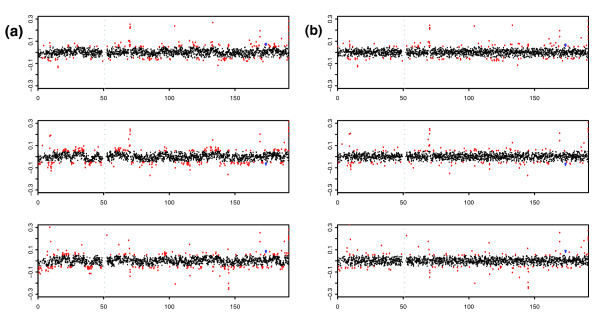
A threshold-based method cannot capture CNV at clone Chr4tp-6G5. The log_2 _ratios for all clones on chromosome 4 are plotted for three samples: NA11829 (top), NA12044 (middle) and NA19093 (bottom) on **(a) **uncorrected and **(b) **corrected data. The log_2 _ratios are plotted on the y-axis and the corresponding genomic position of each clone is plotted on the x-axis. The blue spot flags a clone (Chr4tp-6G5) whose CNV status in this individual had previously been determined using a genotyping algorithm [1]. This clone has been classified as a gain in samples NA11829 and NA19093 and a loss in sample NA12044. All of the points with an absolute log ratio greater than 0.05 are highlighted in red. The vertical gray dashed line indicates the position of the centromere. A threshold sensitive enough to identify this clone results in a large number of clones being flagged (Additional data file 5), many of which almost certainly represent false positives due to confounding with the peaks and troughs of the wave.

**Figure 6:**
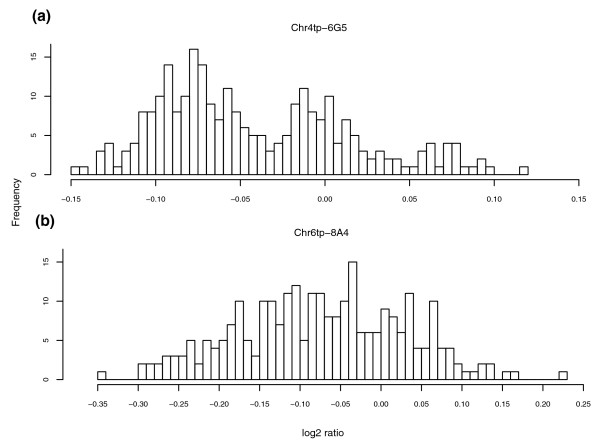
Histogram of the log_2 _ratios for 'grouped' and 'noisy' clones. Histograms of the distribution of the log_2 _ratios for all 269 samples are shown before wave correction for **(a) **clone Chr4tp-6G5 and **(b) **clone Chr6tp-8A4.

Moreover, threshold-based methods are poor at identifying common small CNVs where the log_2 _ratios clearly separate into distinct groups despite their small absolute values. An example of this is a CNV identified and shown to obey Mendelian inheritance in [1] that lies within clone Chr4tp-6G5. Although this clone is more easily distinguished from the background in the wave-corrected data (Figure 5), it remains below the CNVfinder thresholds and so was not called as a CNV when CNVfinder was applied to either corrected or uncorrected data. In principle, making calls on a cross-sample basis better allows the assignment of samples into components that reflect these subtle changes in log_2 _ratio. The clustering of the distribution of log_2 _ratios of the above aCGH clone in the uncorrected WGTP data supports this hypothesis (Figure 6a).

Motivated by this example, we developed a model, CNVmix, that utilizes cross-sample information to call CNVs. Correcting for the wave effect is essential since the variability in the amplitude of the wave across samples is confounded with real copy number changes. By using CNVmix in conjunction with a wave correction, it should be possible to achieve better and more biologically meaningful calls than can be obtained using a threshold-based method. We now give a brief description of CNVmix before applying it to the wave-corrected data.

As well as accounting for the wave artifact, it is also vital to remove other systematic sources of variability between hybridizations to ensure the success of a cross-array calling method since only by doing this is it possible to effectively compare data from different experiments. In CNVmix we did this by adjusting the log_2 _ratios so that the median absolute value was the same for each array. Subsequently, for each clone, CNVmix fits a mixture model [13] to the corresponding log_2 _ratios for all 269 hybridizations. For each clone, the optimal number of components (different copy number states) is identified using a likelihood-based criterion. After identifying the optimal number of components (between 1 and 9) and assigning each sample to the appropriate component, the next problem is to decide which clones harbor CNVs and subsequently which samples are variants for that clone. We do this using a scheme that takes account of the number of components and their means and variances. CNVmix can be thought of as a univariate model in that each clone is treated independently when fitting the mixture model. Of course, it is possible to fit a multivariate mixture model to a number of successive clones [4,14]. This could be useful where a small number of samples (one or two) exhibit slightly higher or lower log_2 _ratios (relative to the remaining samples) over consecutive clones. A univariate model may not detect these changes. However, since CNVs are relatively small [8], the majority should fall within a single clone and so can be identified by a univariate model. Moreover, having fitted a multivariate model, identifying interesting components is difficult due to the (potentially very) complex structure of the fitted model. Despite these problems, extending CNVmix to the multivariate case might offer some potential benefits and we are actively considering the best way that this could be done. Another way in which CNVmix could be developed is to let the components have a non-Gaussian distribution (that is, one with heavier tails). This could reduce the chance of calling false positive clones and, thus, increase the specificity of the model. Finally, the strength of a mixture model approach lies in the classification of samples after a clone has been identified as CNV harboring, and so one could countenance an alternative method for finding such clones to be followed by application of CNVmix to classify individual samples.

### Assessing the performance of CNVmix using validated data

The ideal data for quantifying the performance of CNVmix (indeed any algorithm that uses cross-sample information) would be the unambiguous CNV status of many clones across a large number of samples, determined using an independent method. However, this type of 'gold standard' dataset is not available and so to quantify the performance of CNVmix, we used the validated data described earlier. To use these data to determine how CNVmix performed, we added the five replicate experiments to the HapMap samples and (after correcting for the wave effect) applied CNVmix to the combined dataset. By doing this, we obtained a list of CNV calls for each of the five replicates and estimated the sensitivity, specificity and FDR (Table 3) and compared these with those found using CNVfinder and described earlier (Tables 1 and 2 and Additional data file 6).

**Table 3:** Performance of the CNVmix algorithm using validated (and wave corrected) data

		Calls				
		
Status	Number of regions	A	B	C	D	E
Unvalidated	25	5	5	6	6	5
Validated	112	80	82	83	71	68
Total	137	85	87	89	77	73
						
False discovery rate		6	6	7	8	7
Sensitivity (confidence interval)		71 (61, 79)	73 (64, 81)	74 (65, 82)	63 (54, 72)	61 (51, 70)
Specificity (confidence interval)		80 (61, 92)	80 (61, 92)	76 (57, 90)	76 (57, 90)	80 (61, 92)

The number of regions called in five replicate experiments (A to E, ranked by standard deviation) using CNVmix with the thresholds described in the Materials and methods. The false discovery rate, sensitivity and specificity are quoted as percentages and the confidence intervals quoted are the 95% intervals.

For the replicates with the lower standard deviations (A, B and C) there is a small increase in the FDR (relative to the rates reported in Tables 1 and 2) due to the incorrect calling of one or two more false positive clones (Table 3). However, this is accompanied by an increase in the number of CNVs that are correctly identified, resulting in an increased sensitivity with only a small corresponding decrease in specificity (Table 3). Consequently, for replicates A, B and C there did not seem to be a major difference in the performance of CNVfinder and CNVmix.

However, for the higher variability replicates (D and E) there are more notable differences. Using CNVmix, a small number of additional clones are incorrectly identified (Table 3). However, the number of incorrectly identified clones is no greater than the number identified for replicates A, B and C. Moreover, it is noticeable that despite the higher variability, the sensitivity is still high; this runs in tandem with a relatively high specificity. In contrast, CNVfinder yielded lower estimates of the FDR (Tables 1 and 2) but had much lower sensitivity for these replicates.

As a result, CNVfinder identifies far fewer CNVs than CNVmix for experiments with higher standard deviation. This suggests that as a result of drawing information from across experiments, CNVmix is better than CNVfinder at identifying CNVs when the data vary in noise. Importantly, this improvement is accompanied by only a small increase in the number of unvalidated clones identified relative to the number of such clones called when CNVfinder is applied to the lower variability replicate experiments. Thus, it seems reasonable to conclude that CNVmix can identify CNV clones more consistently than CNVfinder across experiments with differing amounts of noise.

Finally, we note that the comparison described above is somewhat biased against CNVmix since, by design, it will identify some clones where the log_2 _ratios of the samples vary considerably without falling into distinct groups (for example, Chr6tp-8A4 (Figure 6b)), sometimes as a result of the clone containing several independent CNVs. For such clones, individual samples are not called as CNVs. Instead, these clones are flagged as 'complex' by CNVmix and should be thought of as needing more thorough investigation. However, to facilitate the comparison between CNVmix and CNVfinder described above, we called a sample as a CNV (for these clones) if its absolute log_2 _ratio was greater than 0.15. The value of 0.15 was chosen since it is more conservative than that needed to flag an individual component as harboring CNV samples (see Materials and methods) and thus reflects the higher variability associated with such clones. Consequently, in practice the performance of CNVmix is likely to be better than that described above. Therefore, despite the limited amount of validation data available, we believe these results demonstrate clearly the efficacy of CNVmix.

### Calling CNVs in the WGTP dataset using CNVmix

**Figure 7:**
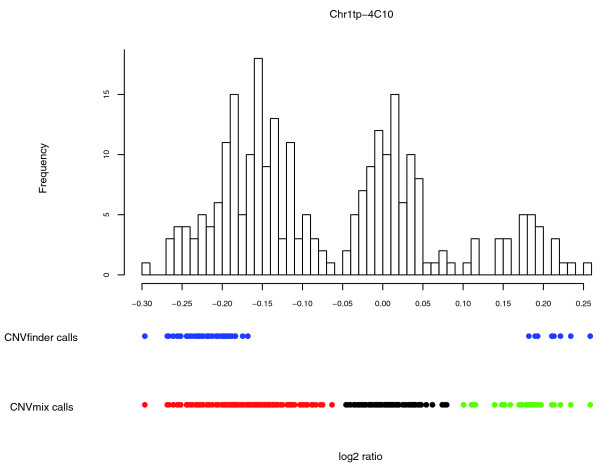
Comparing the calls made by CNVfinder and CNVmix. For clone Chr1tp-4C10, the distribution of the log_2 _ratios analyzed using CNVmix is plotted as a histogram. Blue dots indicate samples called as a CNV for this clone by CNVfinder. The green dots indicate the samples assigned to a copy number gain component, the red dots indicate samples assigned to a copy number loss component and the black dots indicate samples called as normal by CNVmix.

When clones flagged as 'complex' were excluded, CNVmix identified an average of 273 autosomal CNV clones per sample from the wave-corrected data. Moreover, if we use the method described in the previous section for allocating samples into components that represent CNV gain or loss for the 'complex' clones, an average of 334 CNV clones were identified for each sample. By comparison, CNVfinder called an average of 205 CNV clones per sample in the same data. The greater number of CNVmix calls per individual could be due either to greater sensitivity for detecting CNV in a given individual or to calling many more false positive CNVs. The comparison against the Q-PCR validation data described above suggests that the former is more likely. In support of this conclusion, across the entire set of individuals CNVmix identified fewer putatively CNV-harboring clones (2,079) than CNVfinder when applied to either the wave corrected data (2,988) or the uncorrected data (2,392). For more information on the clones identified by CNVmix and whether a sample is called as a CNV for that clone, see Additional data files 7 and 8. In other words, CNVmix identified each putative CNV clone in many more individuals and was better able to assign samples into distinct groups that reflect changes in copy number (Figure 7). We also examined the heritability of the CNV calls made by CNVmix, compared to CNVfinder, by comparing the proportion of CNVs identified in an offspring that were also identified in either parent. Across the 59 offspring present among these samples, the median proportion of CNVs identified by CNVfinder that were also found in either parent was 75.5%, whereas for CNVs identified by CNVmix (excluding the complex CNVs) it was 87.9%. This higher heritability accords with the above inference that CNVmix calls a higher number of CNVs per individual, compared to CNVfinder, due to higher sensitivity, not lower specificity.

After merging overlapping putative CNV clones into CNVRs across the entire set of individuals, CNVmix found slightly more CNVRs (1,271) than CNVfinder (1,181). We further evaluated the quality of the CNVRs identified by CNVmix by examining their overlaps with other sets of CNV calls.

Approximately two-thirds (64%) of the CNVRs identified by CNVmix are also identified by CNVfinder. Moreover, both sets of CNVRs have a similar overlap with other independent CNV datasets (40% and 46% for CNVmix and CNVfinder, respectively), as assessed by overlap with autosomal CNVs in the Database of Genomic Variants, having removed those contributed by the previously published analysis of these data [1]. Note that this last comparison is biased against CNVmix due to the prevalence of CNVs identified using threshold methods in the existing databases.

In summary, CNVmix identifies a similar number of copy number variable regions as CNVfinder with a similar predicted FDR, but because it is more sensitive it identifies many more CNVs per individual experiment.

## Conclusion

We have described the presence of a major technical artifact (a spatially autocorrelated 'wave') that occurs throughout a large, publicly available dataset designed to identify CNVs throughout the human genome. By applying a simple loess correction, we have illustrated that we can significantly improve the clustering of the data into groups that reflect the geographical ancestry of the samples. Additionally, we have demonstrated that current methods for calling CNVs yield more calls when applied to the corrected data. This was accomplished without a major increase in the number of false positive calls. Moreover, the wave correction enabled the development of a novel method, CNVmix, for calling CNVs using cross-sample information. By using CNVmix in conjunction with a wave correction we were better able (for clones identified as harboring CNVs) to categorize samples into groups that represented genuine copy number differences. This led to a large increase in the number of putative CNVs identified per experiment, with no evidence for an appreciably increased FDR.

We (and others) have noted the presence of the wave artifact in a number of aCGH datasets [2], including both clone-based and oligo-based arrays used to identify constitutive and somatic (tumor) CNVs (JCM and MEH, unpublished observations). However, these artifacts have not previously been taken into account in the analysis of such data. For example, Wong *et al*. [15] described a study that used aCGH with a different high-resolution large-insert clone array (the SMRT array) for identifying putative regions of CNV in 95 individuals. When we fitted a loess curve to the processed data described therein and generated heatmaps of the fitted curves (Additional data file 9) we observed that the wave effect was equally striking in this independent set of data. Furthermore, the location of the peaks and troughs of the wave was extremely similar to those seen in the WGTP data. Additionally, we noted that the relatively relaxed CNV calling thresholds described in [15] to identify clones harboring CNVs cut across the peaks and troughs of the wave (Additional data file 10). This further illustrates that wave correction is likely to have a significant effect on the analysis of data from aCGH experiments designed to identify CNVs.

While it is relatively straightforward to correct for the wave effect using loess-based methods when the underlying data can be assumed to have few genuine changes (the situation when trying to identify constitutive CNVs), when there are many large copy number changes (as is often the case with tumor data) using a loess-based method has the potential to remove genuine large rearrangements. Moreover, it is not possible to fit reliably the loess model when analyzing arrays that have low genome coverage (for example, CNV-targeted arrays and low-resolution genome-wide arrays), and it may be necessary to fall back on GC-related correction methods, which we have shown to be partially effective. Hence, we envisage further work by different groups will be needed to develop application-specific approaches for correcting this pervasive artifact.

## Materials and methods

### Fitting the loess curve

After reading the data into *R *[16] we fitted the loess curve to each sample/chromosome using a modified version of the *loessFit *function available within the *limma *software library [17]. Since it is widely known [18] that more variation in log_2 _ratios is seen at the telomeres of chromosomes than at other locations on a chromosome, we added 50 simulated data points at either end of each chromosome for all samples to ensure that the fitted loess curve did not inappropriately smooth log_2 _ratios that reflected genuine biological variation. This data augmentation is also motivated by the known sensitivity of statistical modeling to edge (boundary) effects that result from over-fitting the data at the extremes [19]. The additional points were simulated from a Normal distribution whose mean and standard deviation were taken to be the median and (Median absolute deviation)/4 of the log_2 _ratios for a chromosome/sample. Furthermore, to avoid removing real CNVs we used a window of 50 probes and let all clones with absolute log_2 _ratios greater than 0.3 have a weight of 0 while fitting the loess curve. Moreover, prior information [1] about the location of CNVs that spanned multiple probes was used to assign a weight of 0 to these log_2 _ratios, thus ensuring that large genuine CNVs were not removed; we observed that this principally affected the fit of the loess curve only when CNVs spanned at least 10 clones - this affects only a small minority of clones. We found that fitting a loess curve with these characteristics best removed the wave artifact without eliminating real/potential CNVs.

### Clustering the log_2 _ratios

We clustered the uncorrected and corrected log_2 _ratios using the *hclust *function within the *R *statistical framework [16]. We used a Euclidean distance metric and the Ward agglomeration method [12].

#### CNVmix

To apply CNVmix we used the *R *library *mclust *and the *EMclust *function [13]. This function can be used to fit univariate mixture models where observations assigned to a particular component are assumed to come from a Normal distribution with different means and (potentially) different variances. The optimal model is selected using the Bayesian information criterion. For more details of the method used to fit the model see Additional data file 11 and [13]. The fit of the model was good (when checked manually) for the majority of clones, particularly non-CNV harboring clones and those where the log_2 _ratios fell into distinct components (Figure 7). There were a small number of situations where the model's fit was less satisfactory, but these clones were generally classified as complex. Finally, the model occasionally failed to identify clones where a single sample had a large (absolute) log_2 _ratio and the remaining samples had small ratio values. However, the majority of these clones did seem to be identified. Consequently, we concluded that the fit of the model to these data was good, particularly for the clones that had motivated the model's design.

After fitting mixture models to each clone we identified a clone as one that was likely to harbor a CNV if: one, the range between the means of the fitted components was greater than the 95th percentile of the ranges calculated for all clones; two, the component mean with the largest absolute value was greater than the 95th percentile of this quantity determined across all fitted models; and three, the standard deviation of the log_2 _ratios for a clone and the range between the 95th and 5th percentiles of the log_2 _ratios are both greater than the 95th percentile of these measurements for all clones (this criterion identifies clones where the log_2 _ratios were variable without necessarily falling into distinct components).

If there were no overlap between the clones selected by these criteria, we would identify 15% of the clones as harboring CNVs. However, since some clones will clearly satisfy more than one of the criteria, the actual percentage of clones identified will be lower. While setting these thresholds is relatively arbitrary, they were chosen to ensure that the resulting CNV calls were comparable to previous sets of calls. We assessed a number of different combinations but concluded that none performed better in practice. After using these criteria to identify interesting clones, the next step was to assign all of the samples in a component into one of four categories: normal (0), gain (1), loss (-1) and 'complex' (2). If a clone was called by one of the first two criteria, all of the samples in a component were called as gained/lost if the mean of the component was greater than 0.05 or less than -0.05, respectively. If the clone was called by the third criterion all samples were flagged as 'complex'. Samples in other components were assigned to the normal category. Subsequently, the output was written to two text files: the first contains a list of clones (with their start and end positions) that were called as CNVs and the second indicates whether a sample is flagged as a CNV (0, 1, -1 or 2).

### Determining the confidence intervals

Confidence intervals in Tables 1, 2, 3 were calculated by assuming that the posterior distribution of the sensitivity and specificity followed a beta distribution. The prior distributions were taken as beta(0.5, 0.5) (Jeffrey's prior) [20] in both cases.

#### Availability of code

All analysis was performed using the *R *statistical framework [16] and scripts for implementing the code are available at [21]. We will also incorporate the code in the *snapCGH *[22] Bioconductor library in the near future.

## Abbreviations

aCGH, array comparative genomic hybridization; CEU, Utah residents with ancestry from northern and western Europe; CHB, Han Chinese from Beijing; CNV, copy number variation/copy number variant; CNVR, copy number variable region; FDR, false discovery rate; JPT, Japanese from Tokyo; S:N, signal to noise; WGTP, whole genome tiling path; YRI, Yorubans living in Ibadan, Nigeria.

## Authors' contributions

JCM, NPT and MEH conceived the study and prepared the manuscript. JCM performed the statistical analysis. AV performed the merging of CNVs and applied CNVfinder to the wave corrected data. NPT and MEH directed the study. AGL and ST advised on statistical issues and contributed to writing the manuscript. TDA, TF, RR, HF, BES, ETD and NPC contributed advice and data on biological issues that might explain the wave effect, provided analytical support and contributed to understanding the data.

## Additional data files

The following additional data are available with the online version of this paper. Additional data file 1 shows heatmaps of the fitted loess curve for chromosomes 2 to 22. Additional data file 2 is a comparison of the performance of loess correction and GC linear regression of the WGTP data. Additional data file 3 shows the influence of the dye-labeled nucleotide on the wave pattern. Additional data file 4 shows dendrograms of the uncorrected and corrected log_2 _ratios. Additional data file 5 is a table listing the number of outliers called using a threshold before and after wave correction. Additional data file 6 shows the sensitivity, specificity and FDR for the validated data. Additional data file 7 is a summary of the calls made by CNVmix, giving the ID and genomic location of clones that are called as CNVs. Additional data file 8 is a summary of the calls made by CNVmix, giving details about whether a sample is flagged as a CNV for each of the called clones. Additional data file 9 shows heatmaps of the fitted loess curve for the data described in [15]. Additional data file 10 provides an example of calls in [15] confounded with the wave effect. Additional data file 11 gives details of the CNVmix mixture model.

## Supplementary Material

Additional data file 1 Each page of the PDF file corresponds to an individual chromosome (from 2 to 22). On each page the clones on a chromosome are ordered along the x-axis and the HapMap samples (for samples that are not excluded because of the presence of chromosome-wide gains or losses) are plotted on the y-axis. A green/red region on the heatmap indicates that the fitted loess values in this region are consistently greater/less than zero. The samples have been ordered using the Ward agglomeration method and a Euclidean distance metric. The plot across the top of the heatmap indicates the GC content of each probe and the color bar on the right of the heatmap displays the ethnic origin of a sample: blue (YRI), yellow (CEU) and purple (CHB + JPT). The scale along the bottom of each figure gives the location of the cytobands on a chromosome. Click here for file

Additional data file 2 Comparison of the performance of loess correction and GC linear regression of the WGTP data. Click here for file

Additional data file 3 Three panels display data from two replicate experiments: (A) Fitted loess curves for chromosome 1 (Cy3/Cy5) using dye-labeled nucleotides dCTP (purple) and dUTP (orange); (B) fitted loess curves for chromosome 1 (Cy3/Cy5) in a dye-swap experiment using dye-labeled nucleotides dCTP (purple) and dUTP (orange); (C) smoothed scatterplot of all autosomal dCTP (y-axis) vs dUTP (x-axis) loess fits for the first experiment (Cy3/Cy5); the red line is the regression line fitted to the data, which does not show a negative slope, indicating that changing the dye-labeled nucleotide does not invert the wave effect. Click here for file

Additional data file 4 The top dendrogram (a) shows the clustering of the uncorrected log_2 _ratios for the unrelated HapMap samples for all 22 autosomal chromosomes. The heatbar under the dendrogram indicates the ethnic origin of the sample (blue, YRI; yellow, CEU; purple, CHB + JPT). The second dendrogram/heatbar (b) shows the clustering of the corrected log_2 _ratios on the same chromosomes. Click here for file

Additional data file 5 The columns of the table give the number of clones on chromosome 4 with log_2 _ratios outside a threshold of ±0.06 for the three samples (NA11829, NA12044 and NA19093) shown in Figure 5. The rows indicate the number of clones that are identified using this threshold for uncorrected and corrected log_2 _ratios. A threshold of ±0.06 is necessary in order to identify the red clone that represents a genuine CNV in Figure 5 (log_2 _ratios of 0.071, -0.0665, 0.0835 prior to wave correction, 0.064, -0.069, 0.085 after wave correction). Click here for file

Additional data file 6 The top plot shows the sensitivity (with confidence intervals) for each of the five replicated validation experiments. The experiments are labeled from A to E in order of increasing standard deviation. Lines/points in black represent the sensitivity calculated by CNVfinder on the uncorrected data, lines/points in red represent the sensitivity calculated when CNVfinder was applied to the corrected data and blue lines/points represent the sensitivity calculated when CNVmix was applied to the corrected data. The middle and lower plots show the specificity (with confidence intervals) and FDR, respectively, for the same experiments. The annotation and color scheme is the same as described above. Click here for file

Additional data file 7 The ID and genomic location of clones that are called as CNVs. Click here for file

Additional data file 8 Details about whether a sample is flagged as a CNV for each of the called clones (-1 = deletion, 1 = gain, 2 = complex and 0 = normal). Click here for file

Additional data file 9 Each page of the PDF file corresponds to an individual chromosome. On each page the clones on a chromosome are ordered along the x-axis and the 95 samples that were investigated for CNV in [15] are plotted on the y-axis. (Note that we could not obtain mapping information for 1% of the clones and so they were removed from our analysis.) A green/red region on the heatmap indicates that the fitted loess values in this region are consistently greater/less than zero. The samples have been ordered using the Ward agglomeration method and a Euclidean distance metric. The scale along the bottom of each figure gives the location of the cytobands on a chromosome. Note that some of the heatmaps are predominantly red (noticeably chromosomes 19 and 22) - this is because the median log_2 _ratio is consistently less than 0 for these chromosomes. Click here for file

Additional data file 10 Each page of the PDF contains a plot of the log_2 _ratios for clones (censored at ±0.3) on the long arm of chromosome 7 for five samples analyzed in [15] (samples S20, S30, S32, S40 and S60). The fitted loess curve for this genomic region has been overlaid in blue and the thresholds used in [15] to identify clones harboring CNVs are shown by horizontal dashed gray lines. On all five plots we can observe that the fitted loess curve has a trough at around 75 and 100 Mb (this is common to all samples - see Additional data file 6), suggesting that this is a technical artifact. Moreover, in all five plots a small number of clones in these regions have log_2 _ratios that are lower than the threshold and, consequently, they are flagged (almost certainly incorrectly) as harboring a CNV. Click here for file

Additional data file 11 Details of the CNVmix mixture model. Click here for file
