# Supplementary material for: Breaking the waves: improved detection of copy number variation from microarray-based comparative genomic hybridization
Source: Genome Biol. 2007 Oct 25;8(10):R228. doi: 10.1186/gb-2007-8-10-r228 (PMC2246302; doi:10.1186/gb-2007-8-10-r228)

Chromosome 2

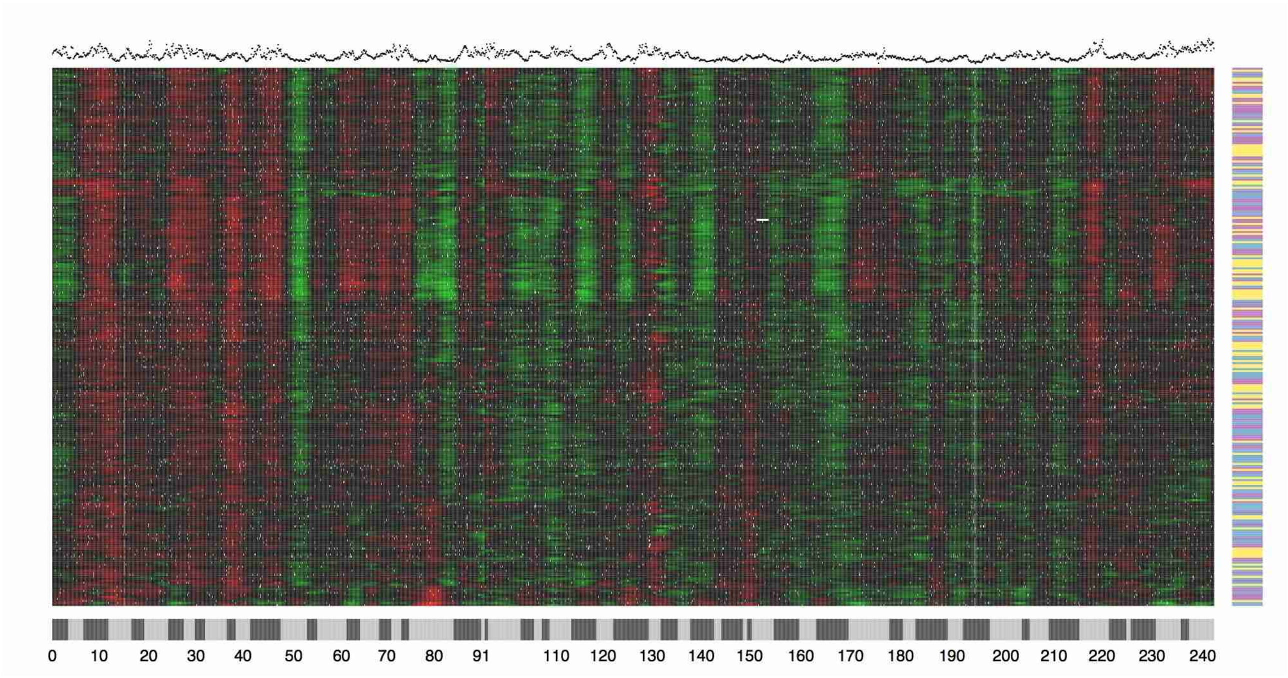

Chromosome 3

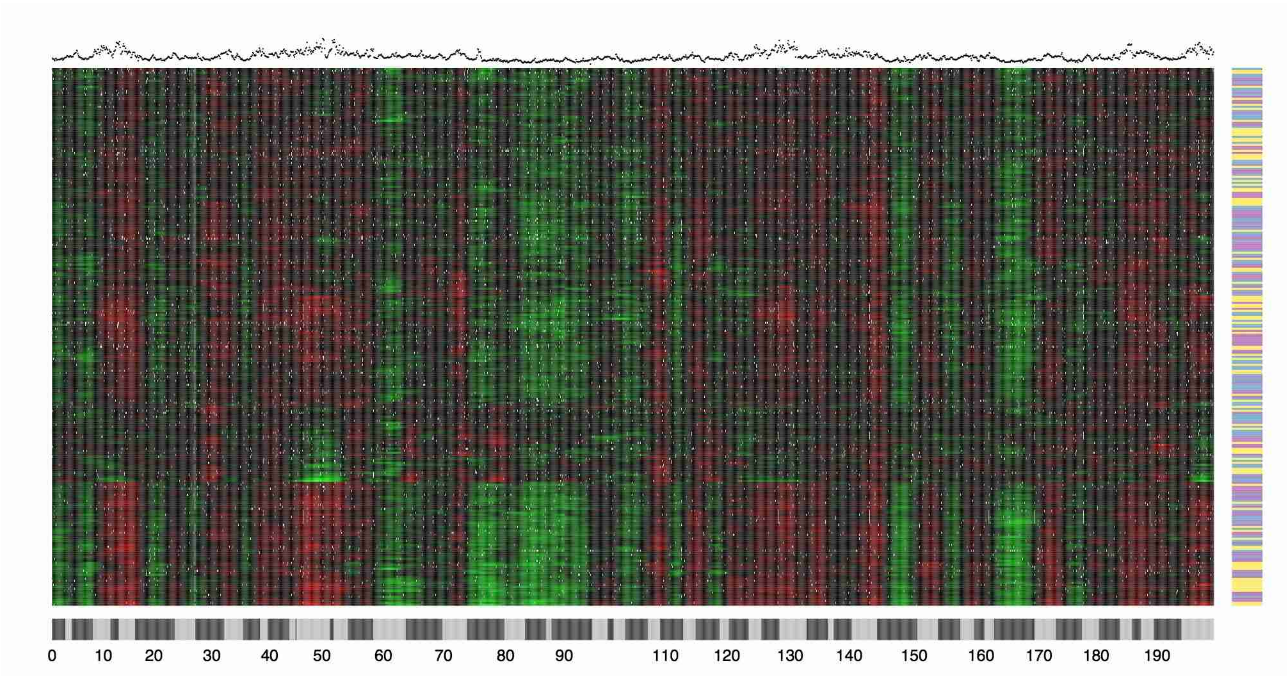

Chromosome 4

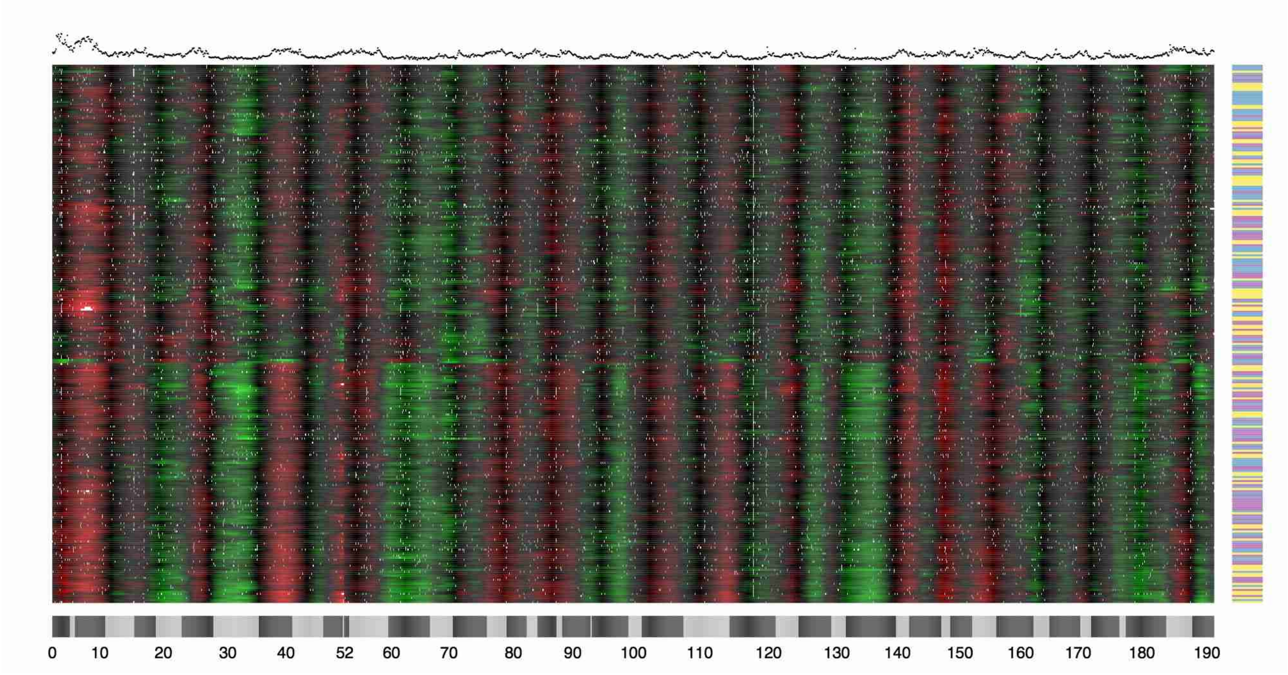

Chromosome 5

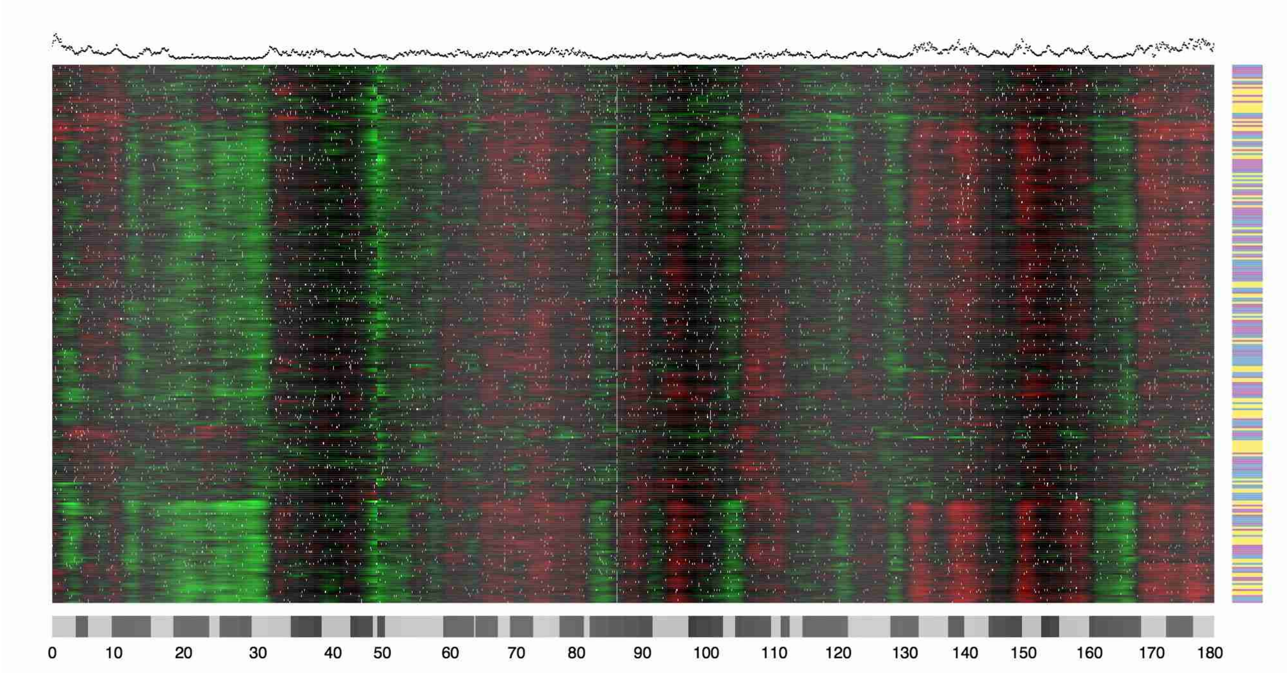

Chromosome 6

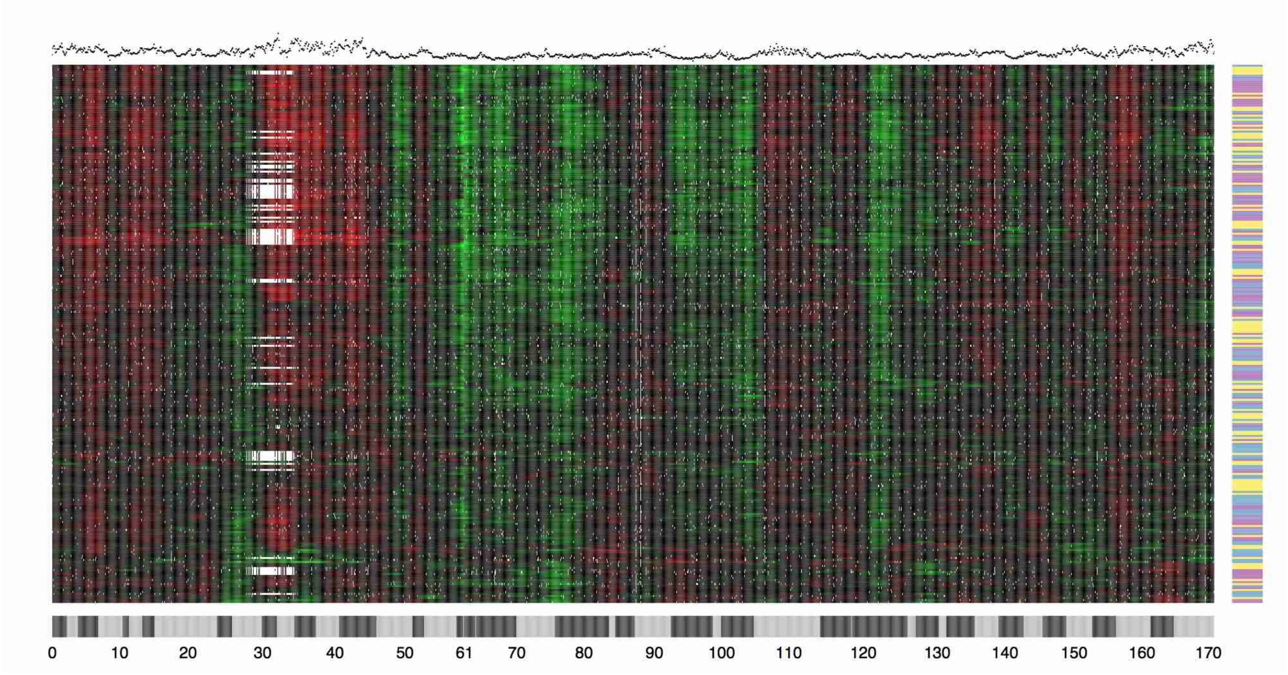

Chromosome 7

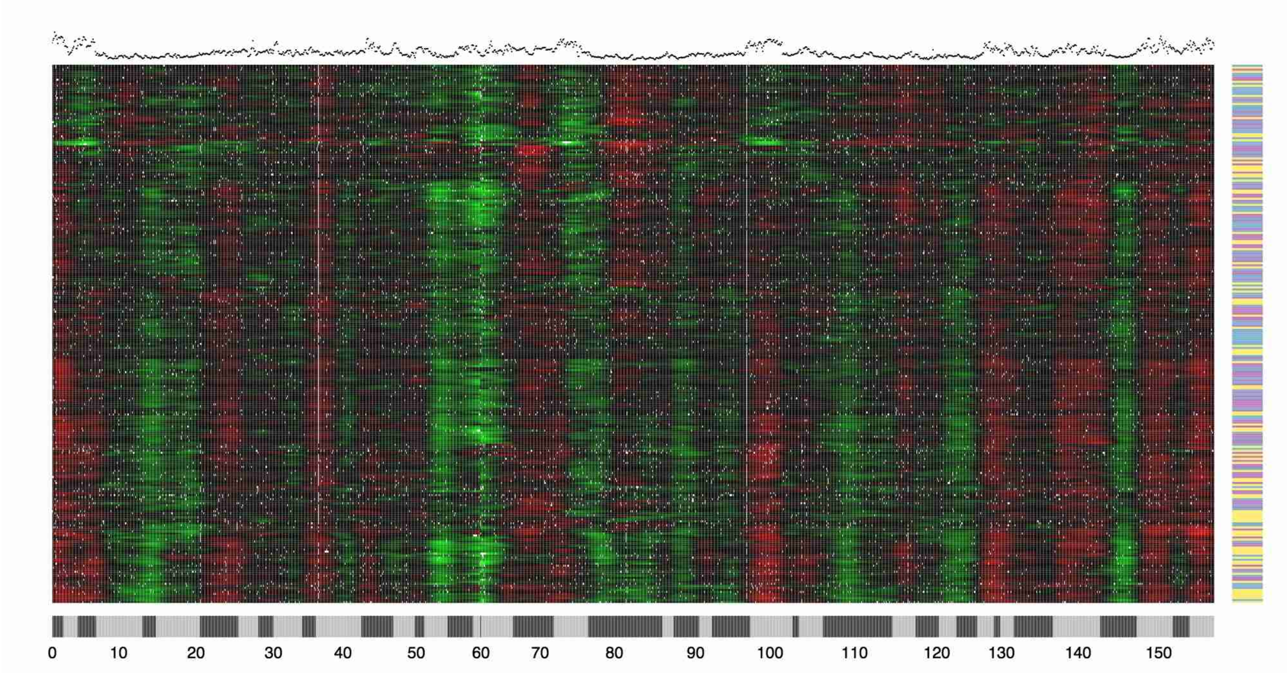

Chromosome 8

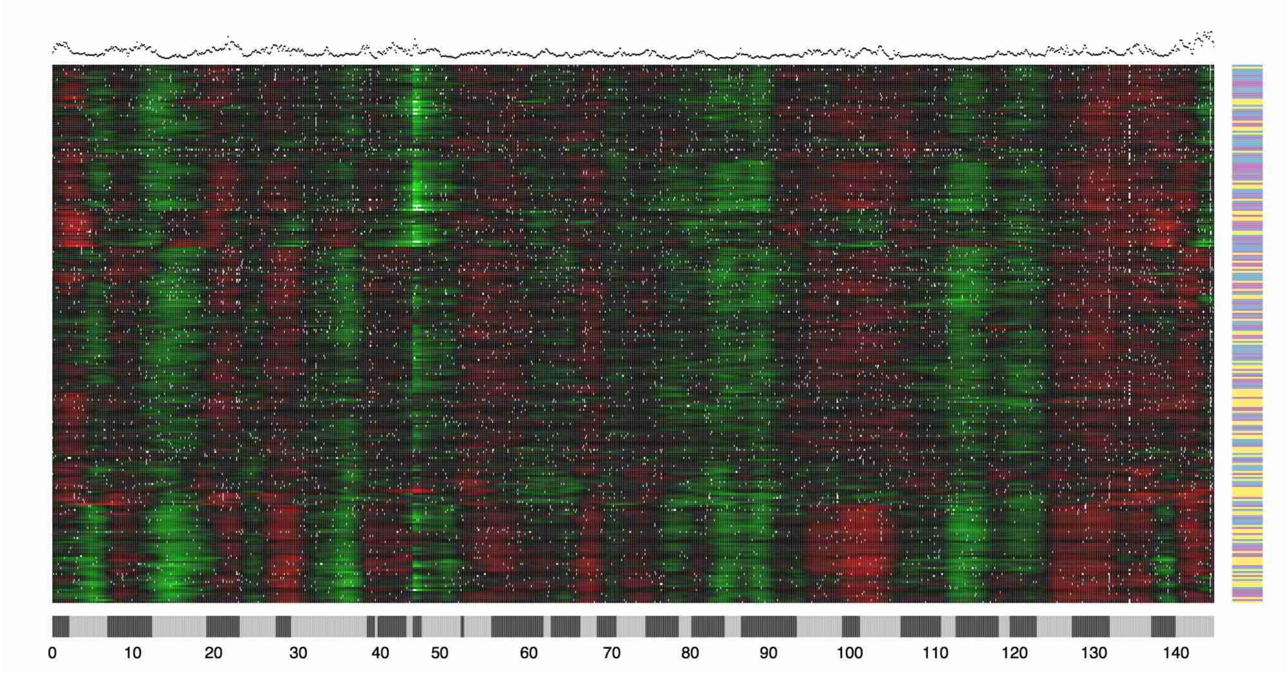

Chromosome 9

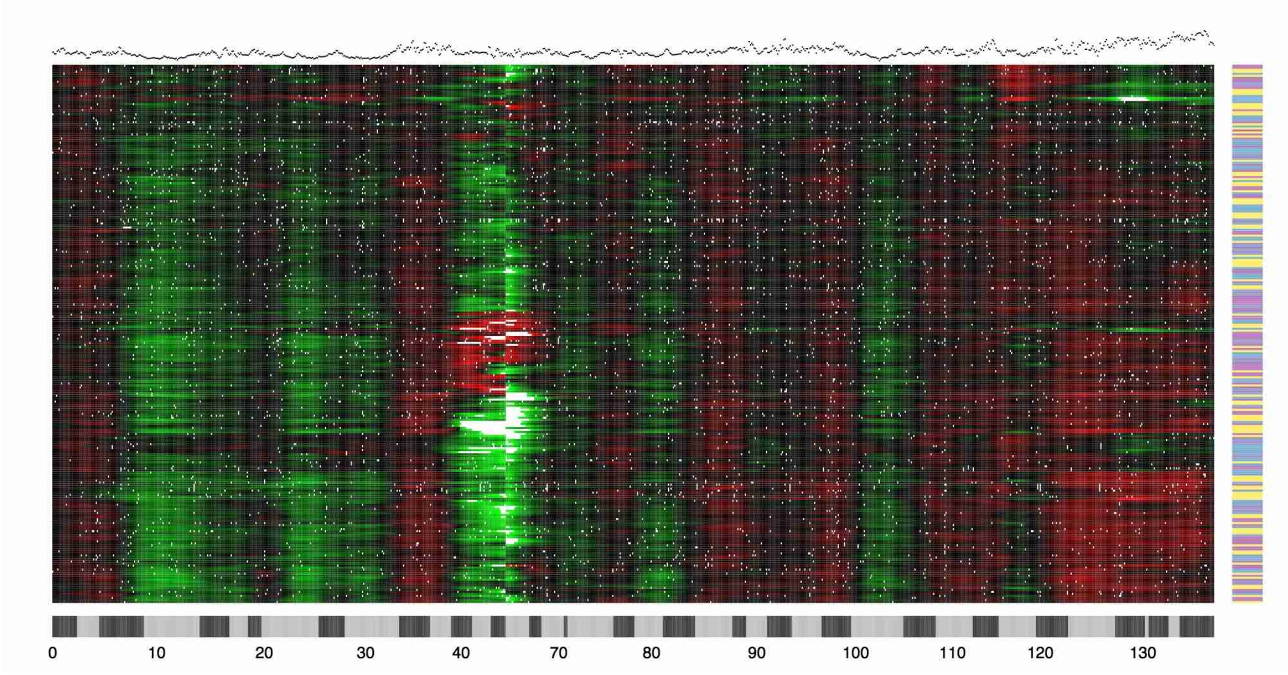

Chromosome 10

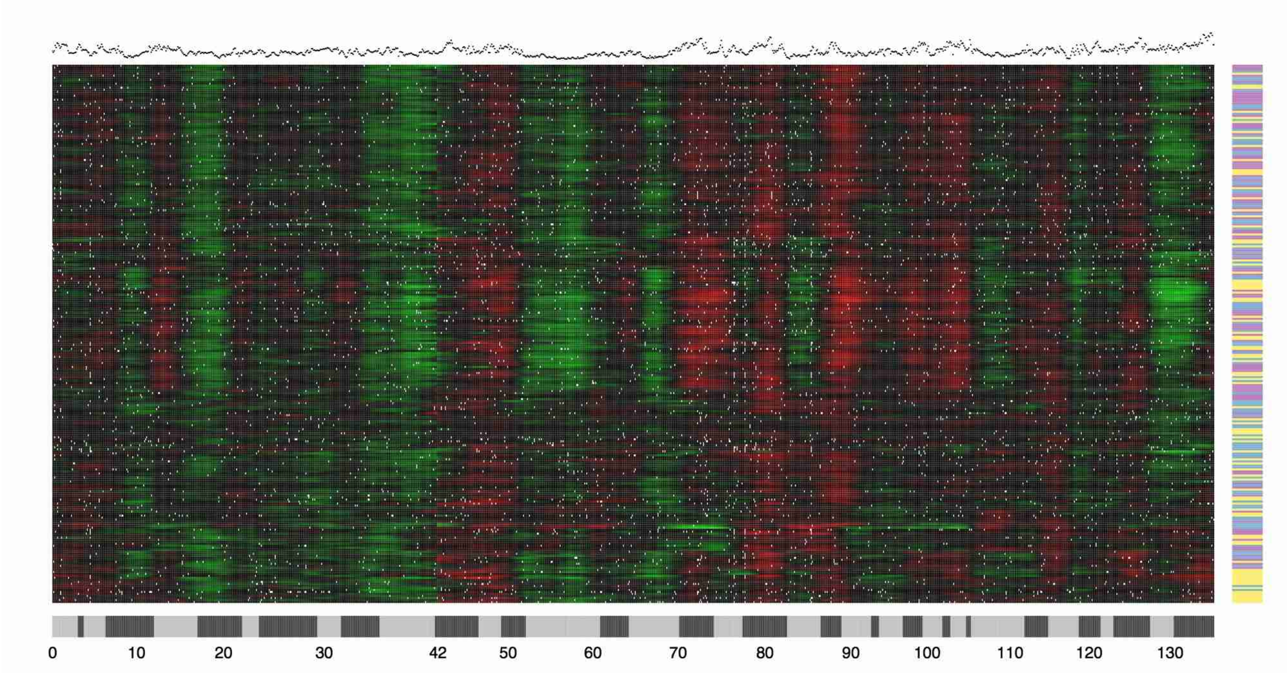

Chromosome 11

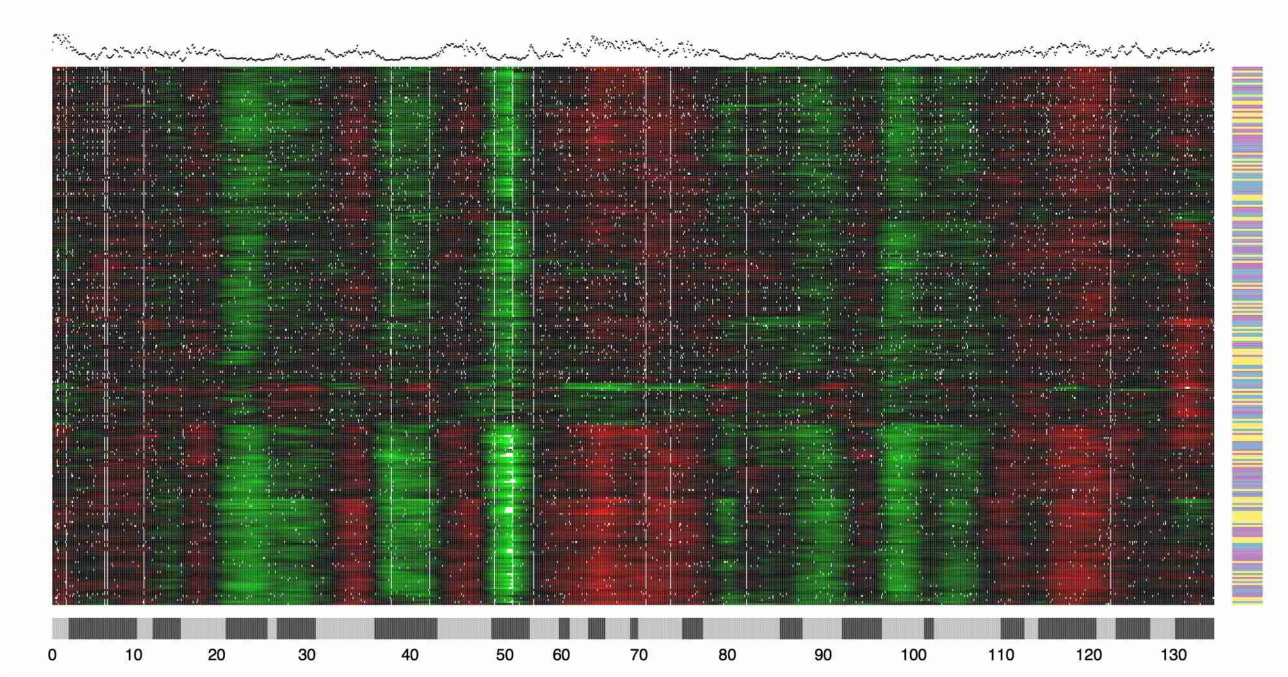

Chromosome 12

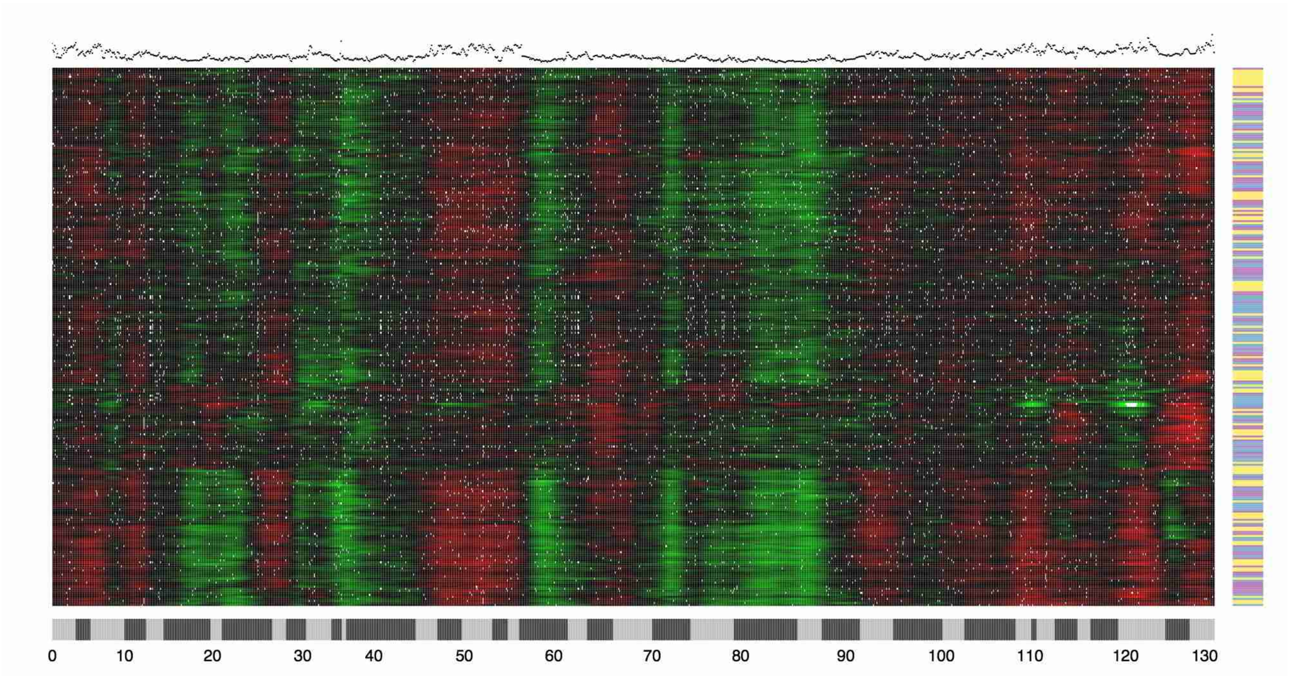

Chromosome 13

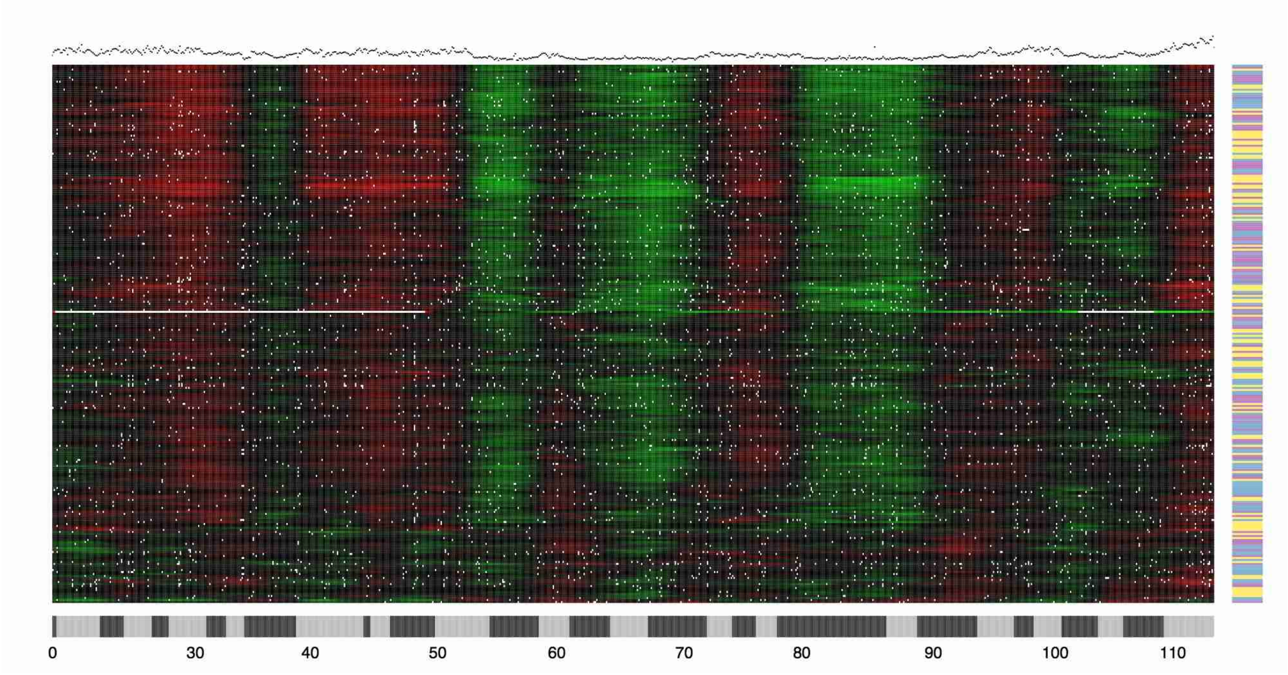

Chromosome 14

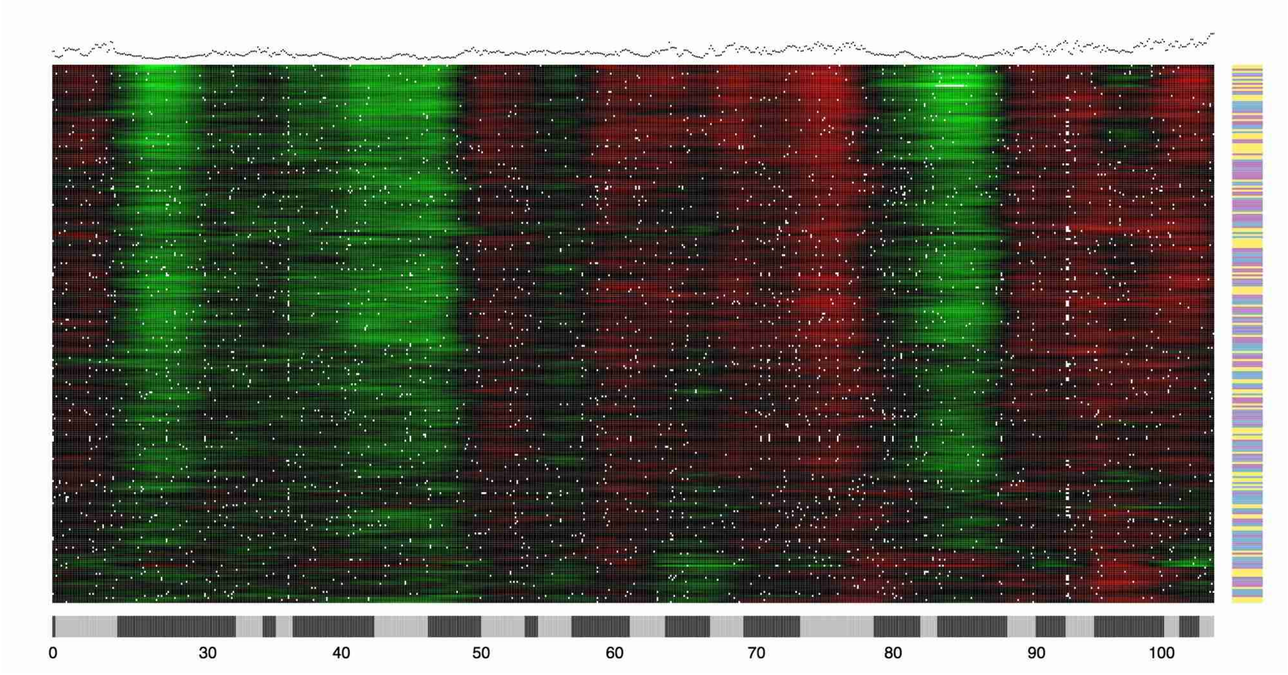

Chromosome 15

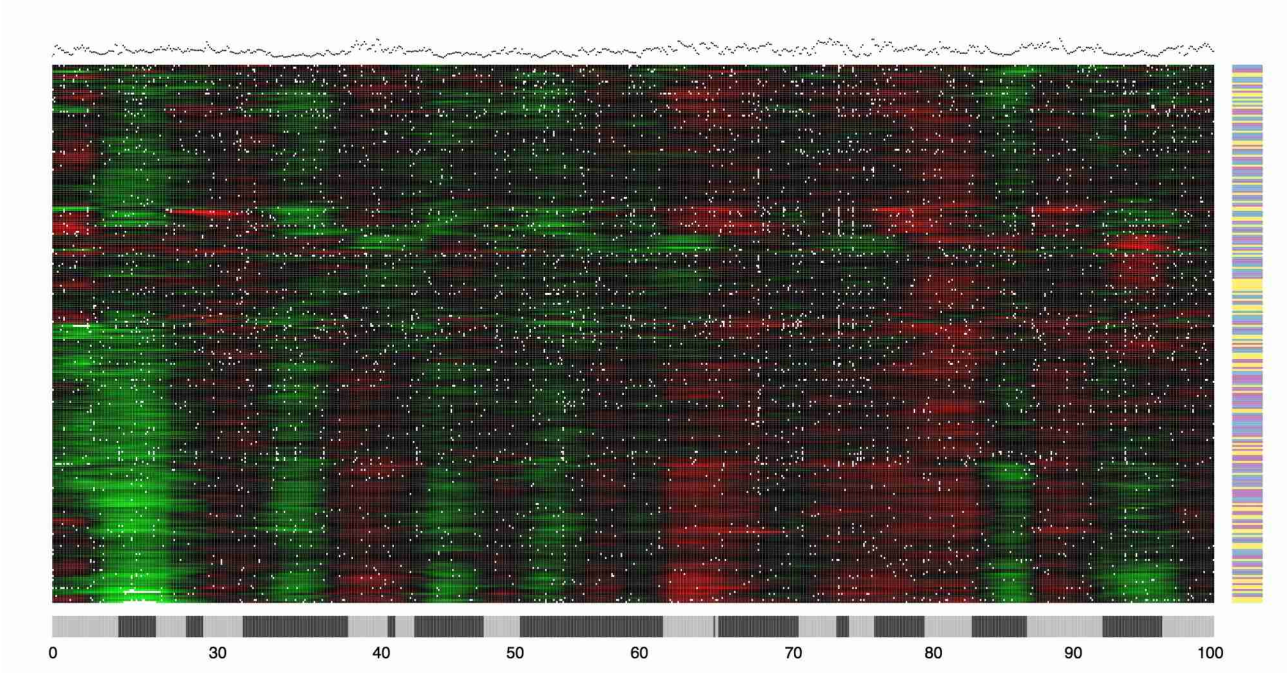

Chromosome 16

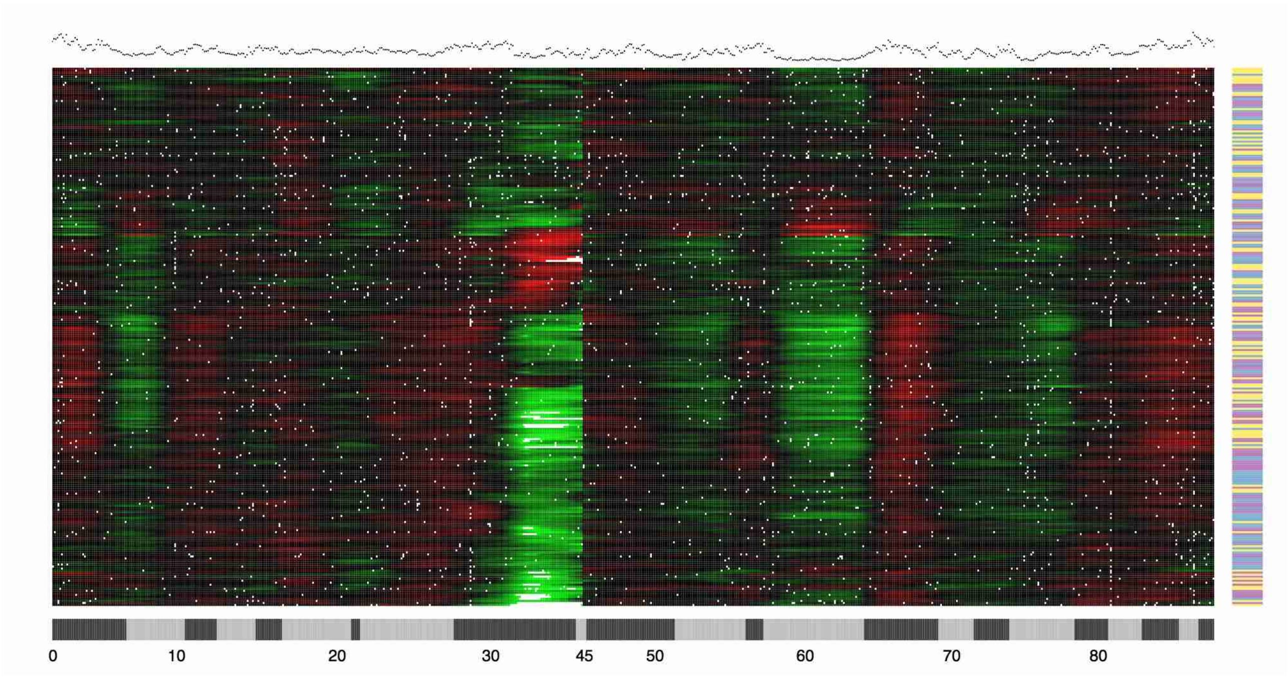

Chromosome 17

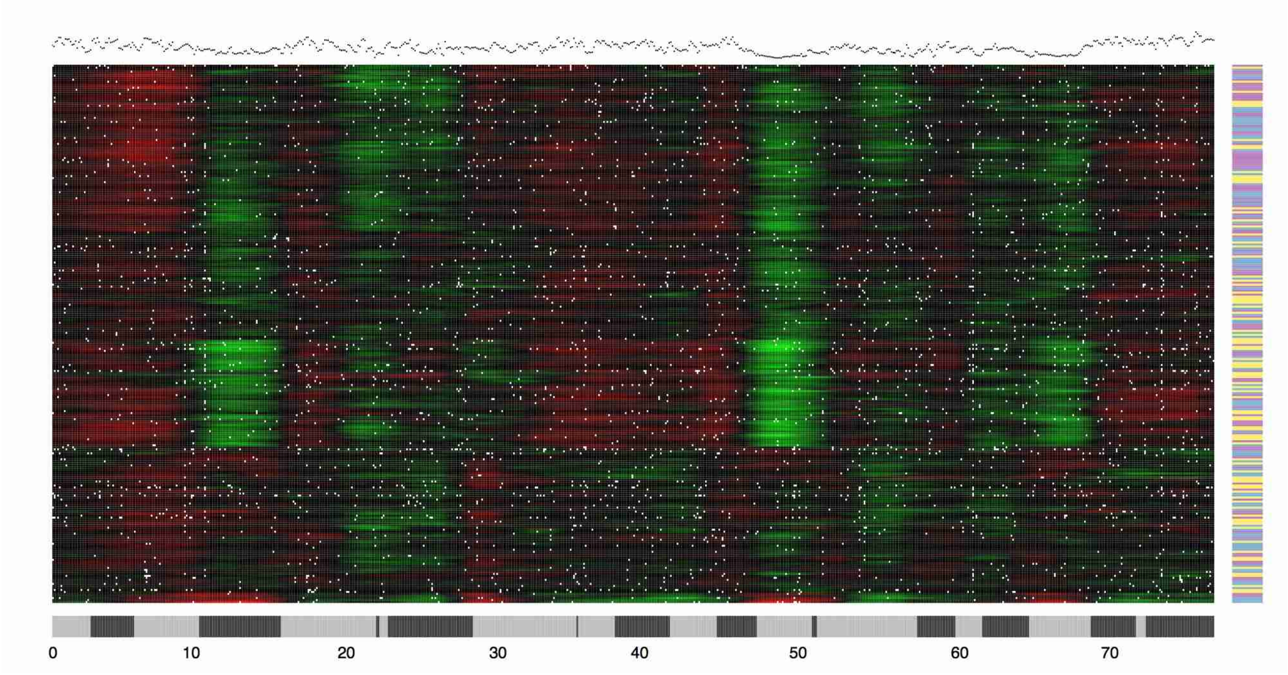

Chromosome 18

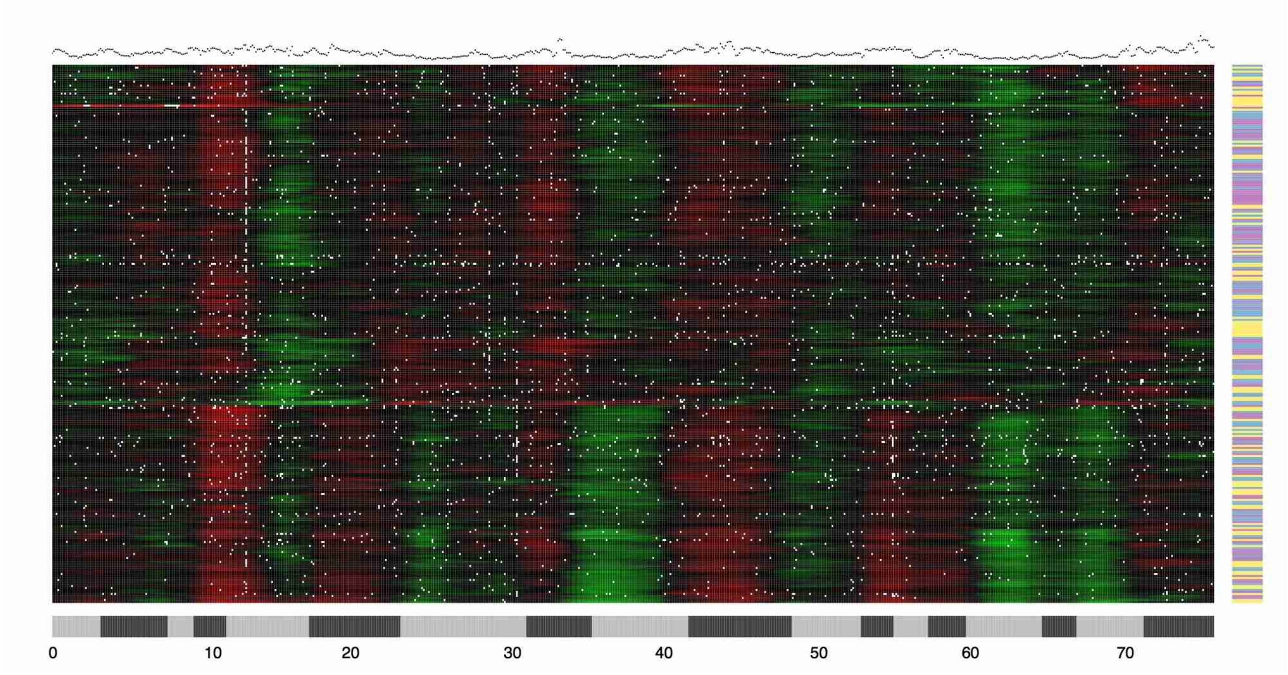

Chromosome 19

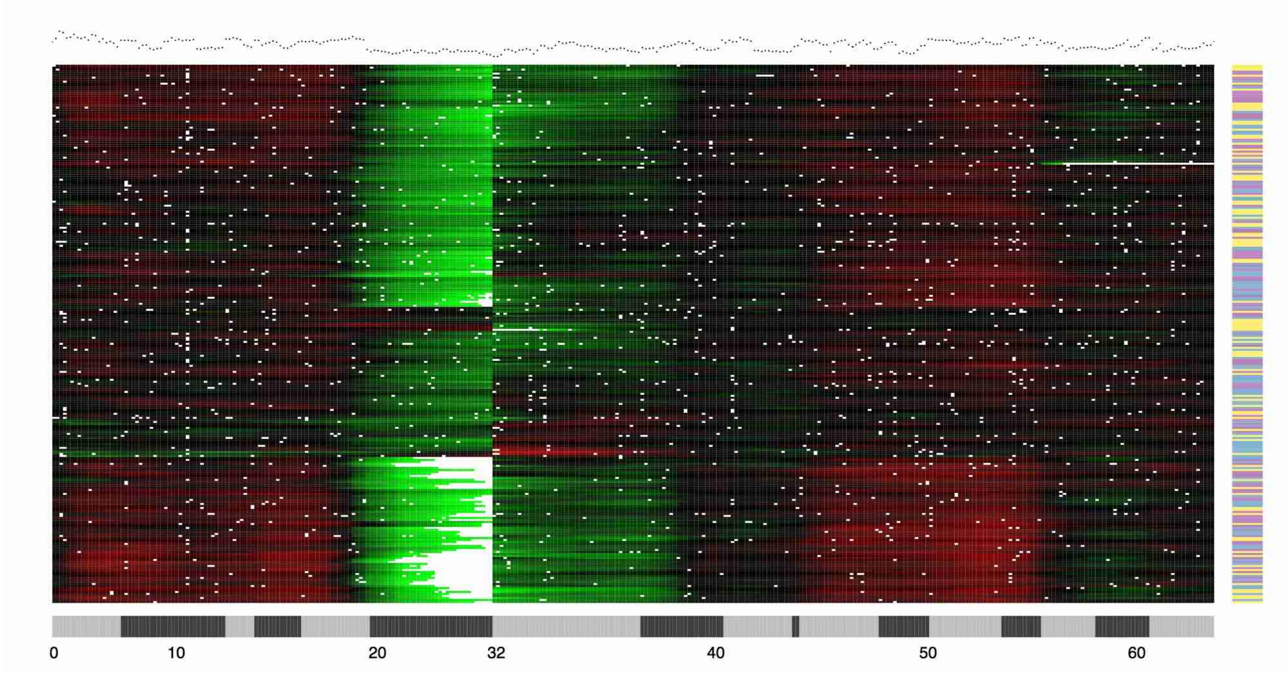

Chromosome 20

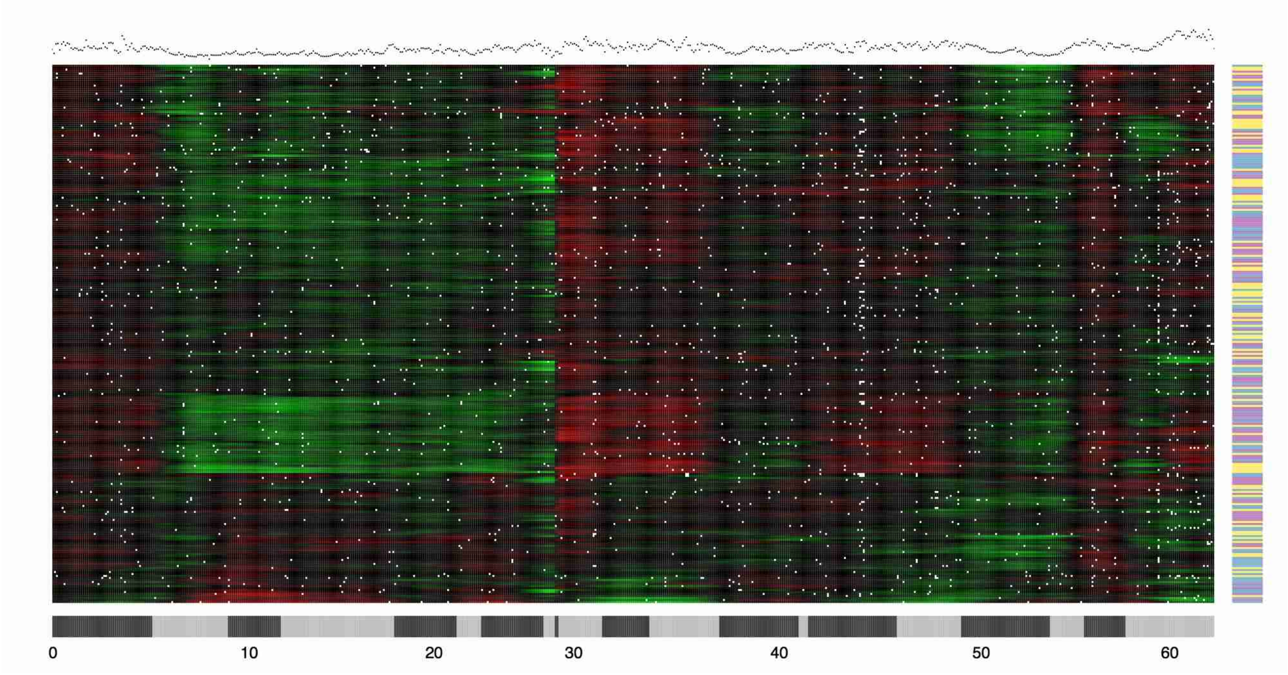

Chromosome 21

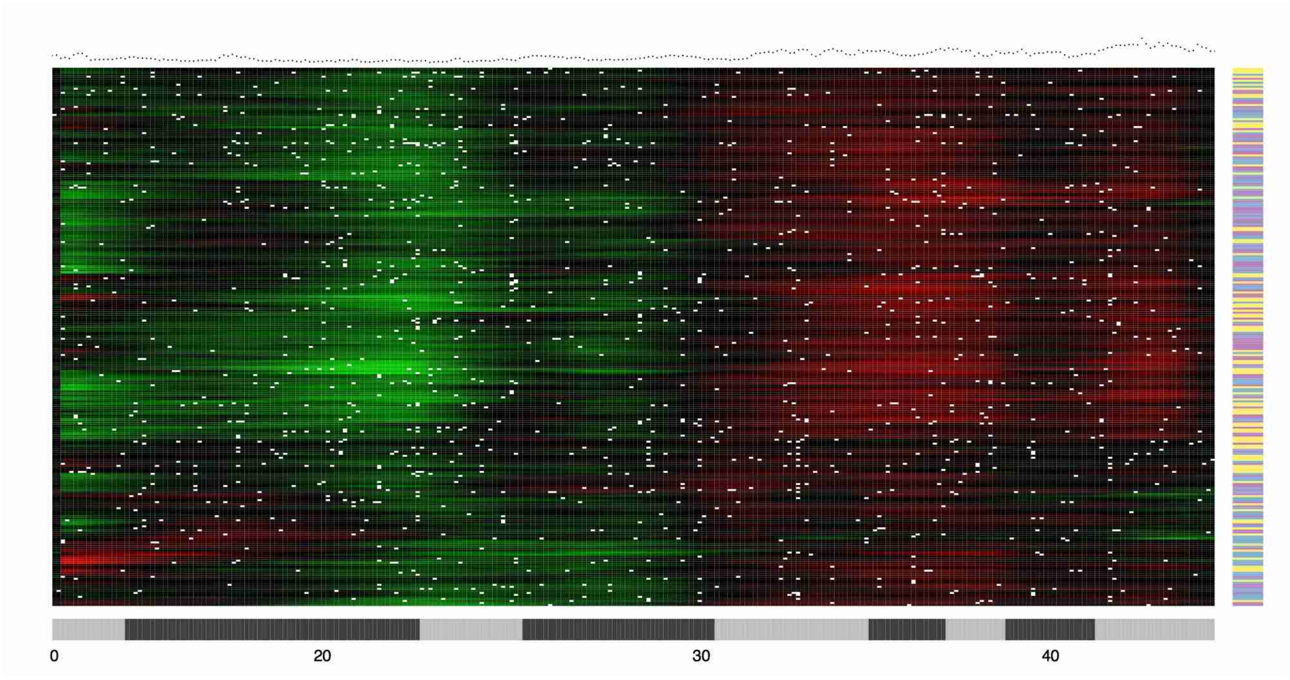

Chromosome 22

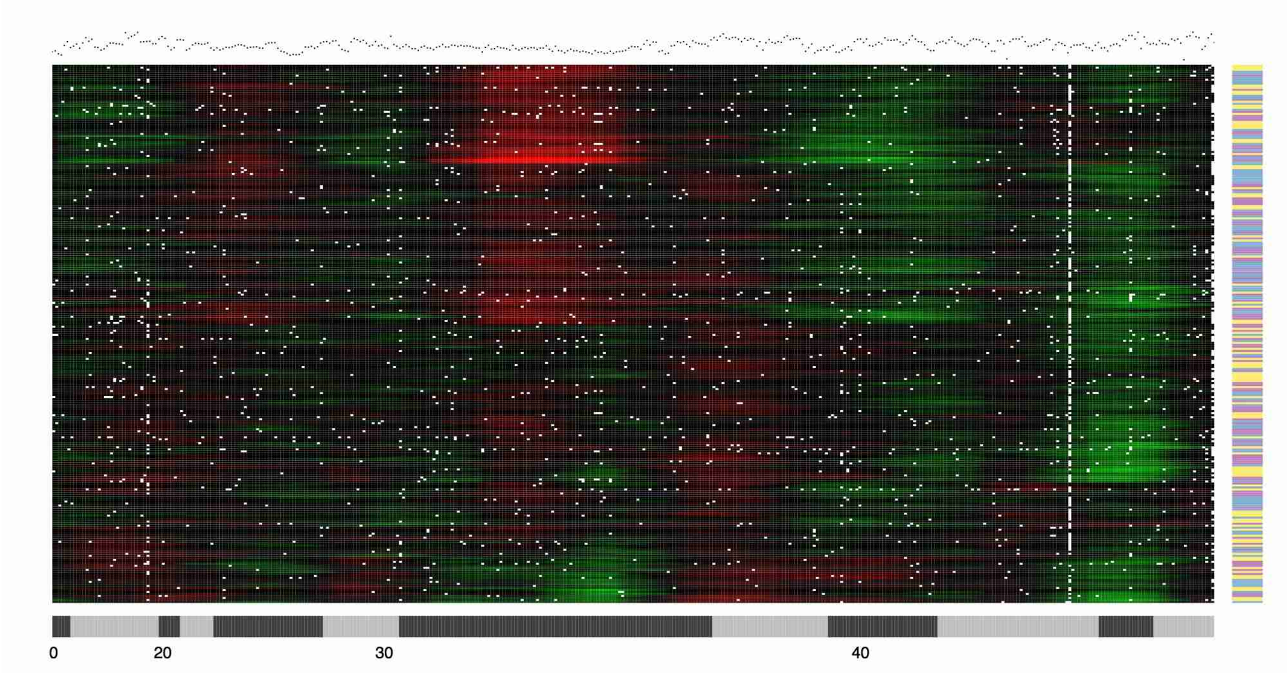

Supplement: Additional data file 1 — Each page of the PDF file corresponds to an individual chromosome (from 2 to 22). On each page the clones on a chromosome are ordered along the x-axis and the HapMap samples (for samples that are not excluded because of the presence of chromosome-wide gains or losses) are plotted on the y-axis. A green/red region on the heatmap indicates that the fitted loess values in this region are consistently greater/less than zero. The samples have been ordered using the Ward agglomeration method and a Euclidean distance metric. The plot across the top of the heatmap indicates the GC content of each probe and the color bar on the right of the heatmap displays the ethnic origin of a sample: blue (YRI), yellow (CEU) and purple (CHB + JPT). The scale along the bottom of each figure gives the location of the cytobands on a chromosome. [file gb-2007-8-10-r228-S1.pdf]
