# Supplementary material for: Breaking the waves: improved detection of copy number variation from microarray-based comparative genomic hybridization
Source: Genome Biol. 2007 Oct 25;8(10):R228. doi: 10.1186/gb-2007-8-10-r228 (PMC2246302; doi:10.1186/gb-2007-8-10-r228)

## Comparing GC regression with loess correction

The aim of the following analysis is to establish whether correcting for the wave effect in the WGTP array data using a GC regression removes the artefact more efficiently than a loess-based approach as described in the paper.

Firstly, we describe how we used the GC content of each probe to correct for the artefact. For each sample and chromosome independently, we fitted a linear regression of the log_2_ ratios on the GC content of each probe. More formally:

y_ijk_ = α_ij_ + β_ij_ x_ijk_ + ε_ijk_

where y_ijk_ is the log_2_ ratio for the k^th^ clone for the j^th^ chromosome for sample i and x_ijk_ is the GC content (as a percentage) of the k^th^ clone for the j^th^ chromosome for sample i. α_ij_ and β_ij_ are coefficients to be estimated and ε_ijk_ is an error term. Subsequently, we defined the residuals from the fitted model as the GC-corrected log_2_ ratios i.e.

y^GC^_ijk_ = y_ijk_ – y^fit^_ijk_

where y^fit^_ijk_ is the fitted value for clone k on chromosome j for sample i.

Subsequently, for each sample and chromosome we calculated the standard deviation of the GC-corrected log ratios and compared them with the standard deviation of the loess-corrected log ratios (for more detail see the paper). For the overwhelming majority of chromosomes/samples (93%) the standard deviation is lower when we apply a loess-based correction to the log_2_ ratios suggesting that, for the WGTP array data, the loess-based approach removes more variation. More precisely, the loess correction method reduces the standard deviation on each sample/chromosome by an additional 3.8% relative to the GC regression based approach. To ensure that this reduction is not due to the additional complexity inherent in the loess-based model we also calculated the AIC on a sample/chromosome specific basis to compare the performance of the two wave correction approaches. Assuming the residuals are normally distributed, the AIC is defined as:

AIC = nlog(RSS/n) + 2 (# parameters) + constant

Here n is the number of observations, RSS is the residual sum of squares and # parameters is the number of parameters fitted by the model. For the GC regression the number of parameters is always 2, while for the loess-based approach an “equivalent” number of parameters can be approximated [23,24] using the loess function within R [16]. Note that it is possible to calculate exactly the degrees of freedom (and hence the number of parameters) but this is computationally intensive^1^ and, from our experience (JCM) seemed to yield only marginally different results from the approximation-based approach. Hence, we decided to estimate the number of parameters using the latter method. In order to compare the performance of the GC and loess based corrections we calculated:

*diff* = AIC_loess – AIC_GC.

If *diff* is less than 0 then, even taking account of its additional complexity, the loess model still explains more of the variation in the data than the GC regression approach.

When we examined the distribution of *diff* we discovered that it was indeed significantly less than 0 (Wilcoxon test, p-value = 3 x 10^-11^). Interestingly, boxplots of *diff* generated on a chromosome-by-chromosome basis (Figure) showed it tended to be more negative (i.e. the loess method performed better) for longer chromosomes. However, with the possible exception of one or two of the shorter chromosomes (particularly chromosome 19 where the coverage of the clones is not as high as the rest of the genome and also for chromosomes 17 and 20) the loess-based method always seems to perform as well, if not better, than the GC-regression based approach. Moreover, even for those chromosomes where the GC-regression seems to have a better performance, the differences in AIC are small relative to those chromosomes where the loess-based approach performs better. Hence, this supports our approach of applying a loess-based correction for BAC-based WGTP array CGH data to correct for the wave artefact.

Figure. Boxplots of *diff* for each autosome. The difference in the AIC (AIC_loess – AIC_GC) for each sample/chromosome is plotted on the y-axis and boxplots of the difference for each chromosome are plotted on the x-axis. The horizontal red line has an intercept of 0. If *diff* is above this line AIC_GC is less than AIC_loess and hence, after accounting for the number of parameters required to fit each model, the GC-based approach out-performs the loess correction.


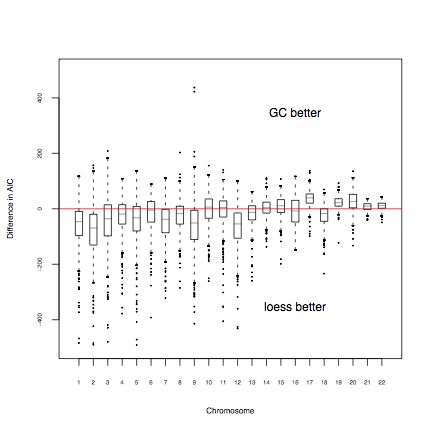

Supplement: Additional data file 2 — Comparison of the performance of loess correction and GC linear regression of the WGTP data. [file gb-2007-8-10-r228-S2.doc]
