# Supplementary figures and images for: Breaking the waves: improved detection of copy number variation from microarray-based comparative genomic hybridization
Source: Genome Biol. 2007 Oct 25;8(10):R228. doi: 10.1186/gb-2007-8-10-r228 (PMC2246302; doi:10.1186/gb-2007-8-10-r228)

A

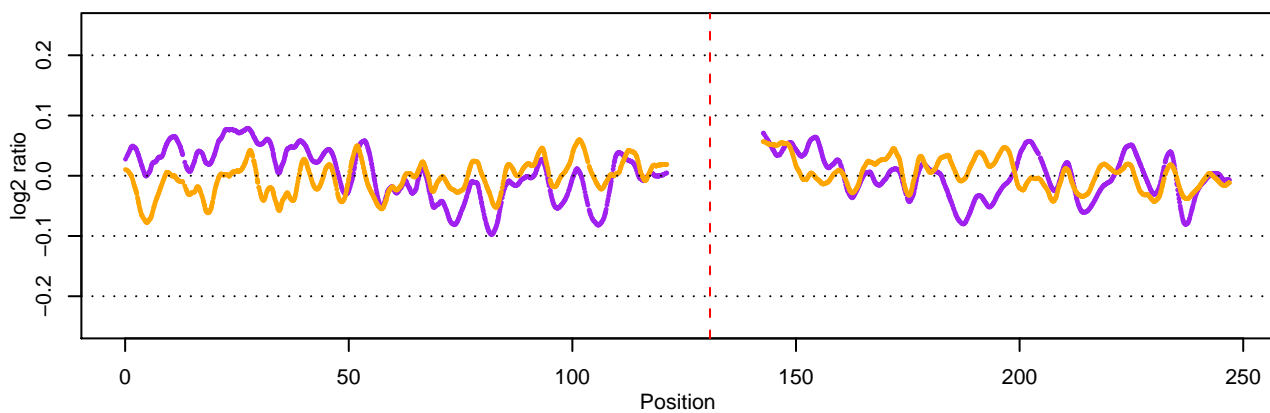

B

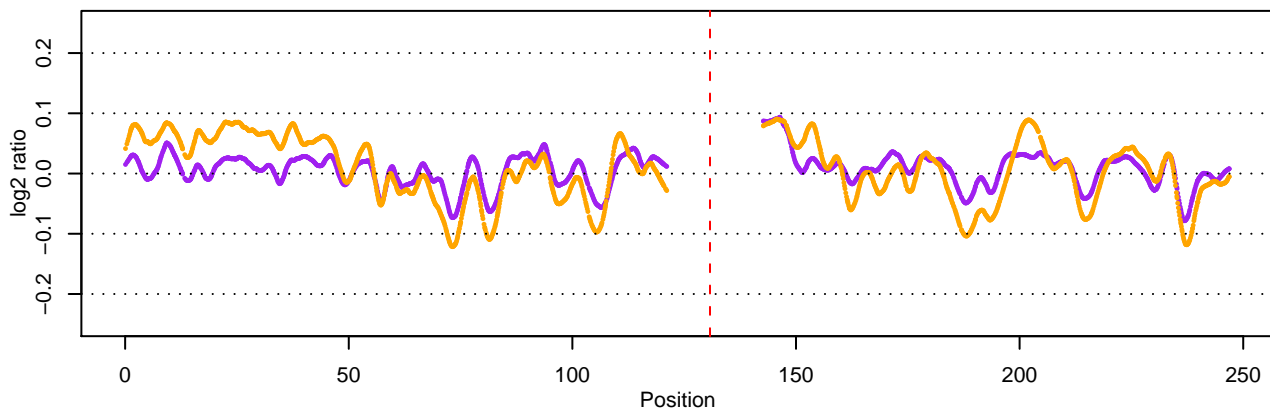

C

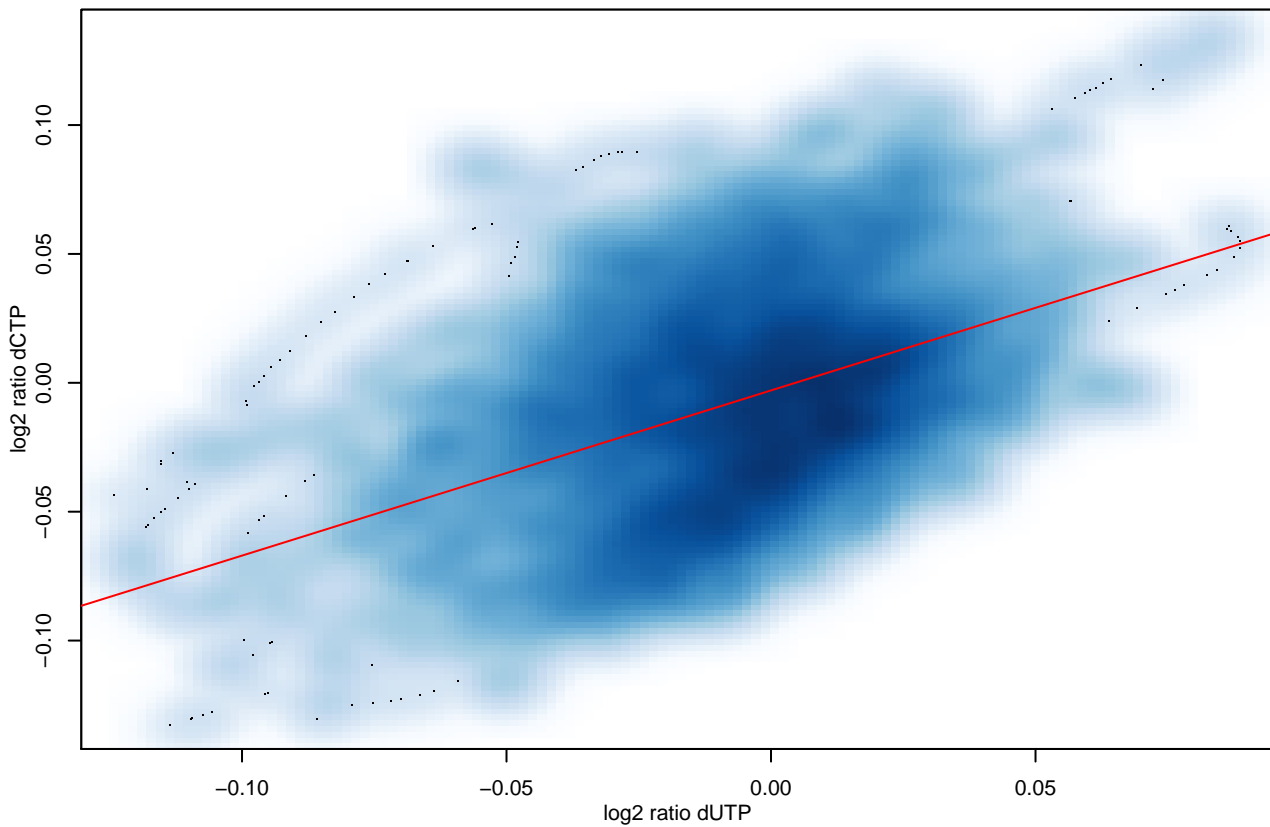

Supplement: Additional data file 3 — Three panels display data from two replicate experiments: (A) Fitted loess curves for chromosome 1 (Cy3/Cy5) using dye-labeled nucleotides dCTP (purple) and dUTP (orange); (B) fitted loess curves for chromosome 1 (Cy3/Cy5) in a dye-swap experiment using dye-labeled nucleotides dCTP (purple) and dUTP (orange); (C) smoothed scatterplot of all autosomal dCTP (y-axis) vs dUTP (x-axis) loess fits for the first experiment (Cy3/Cy5); the red line is the regression line fitted to the data, which does not show a negative slope, indicating that changing the dye-labeled nucleotide does not invert the wave effect. [file gb-2007-8-10-r228-S3.pdf]

a

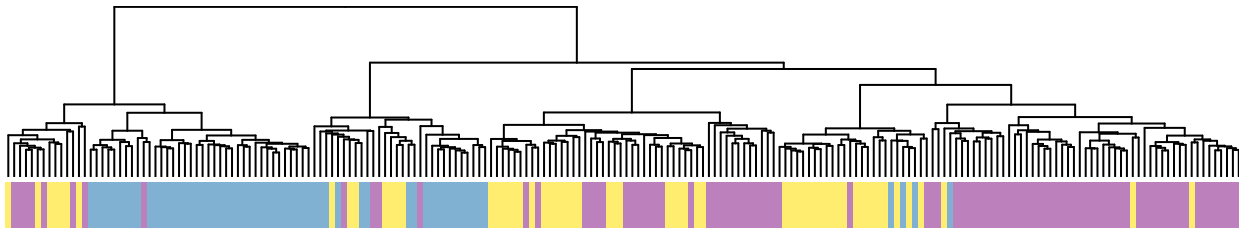

b

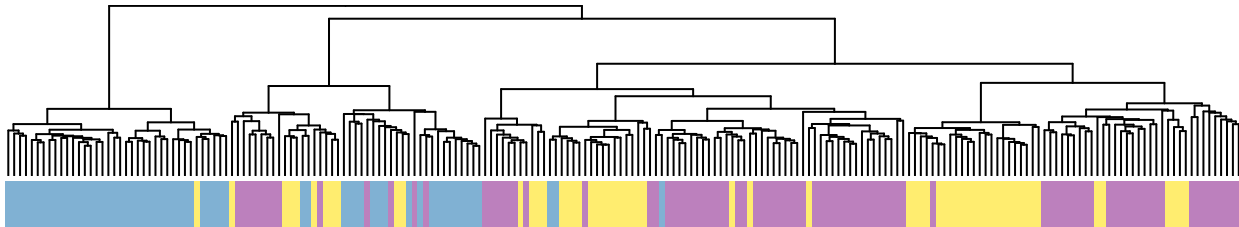

Supplement: Additional data file 4 — The top dendrogram (a) shows the clustering of the uncorrected log2 ratios for the unrelated HapMap samples for all 22 autosomal chromosomes. The heatbar under the dendrogram indicates the ethnic origin of the sample (blue, YRI; yellow, CEU; purple, CHB + JPT). The second dendrogram/heatbar (b) shows the clustering of the corrected log2 ratios on the same chromosomes. [file gb-2007-8-10-r228-S4.pdf]

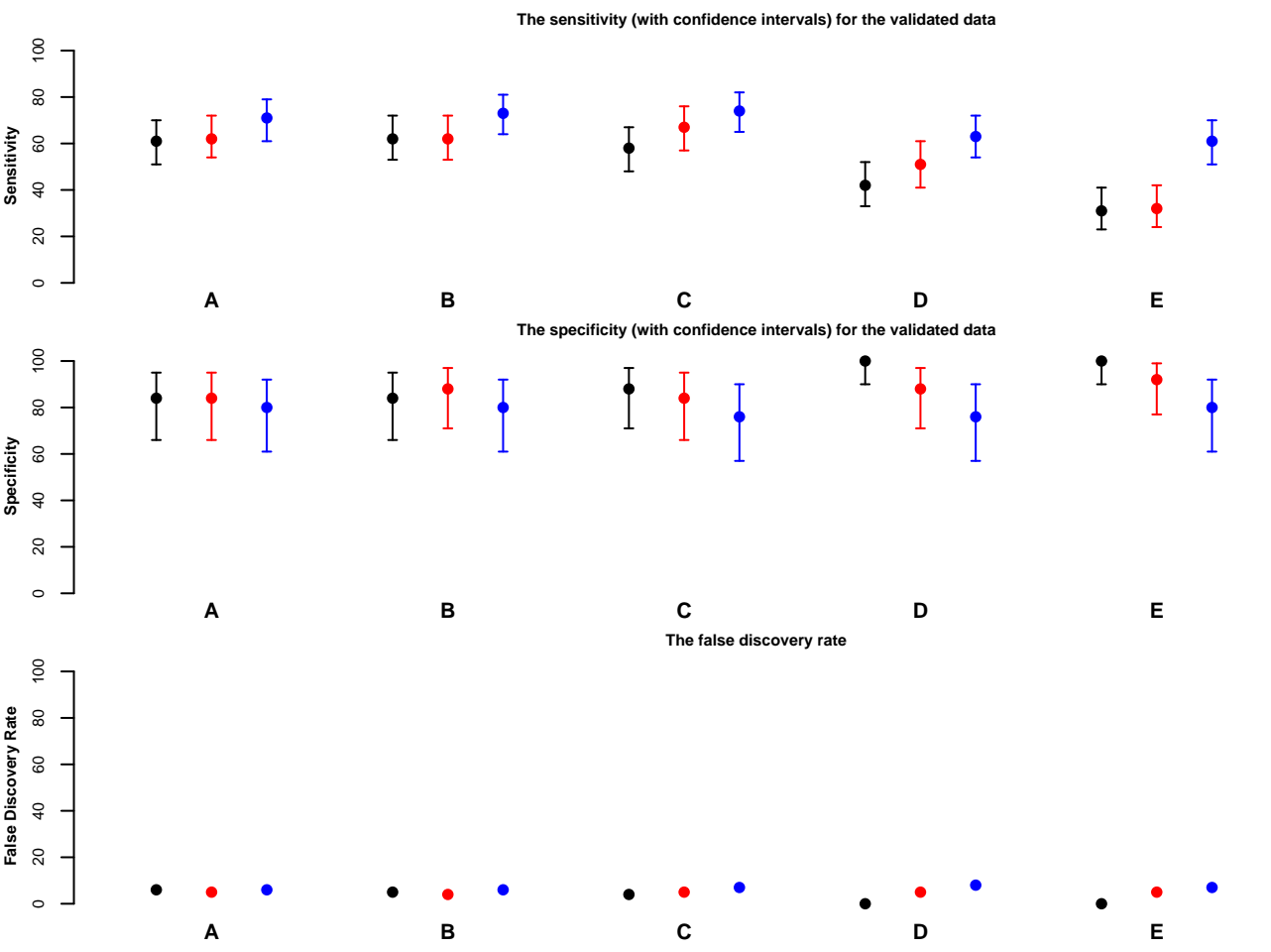

Supplement: Additional data file 6 — The top plot shows the sensitivity (with confidence intervals) for each of the five replicated validation experiments. The experiments are labeled from A to E in order of increasing standard deviation. Lines/points in black represent the sensitivity calculated by CNVfinder on the uncorrected data, lines/points in red represent the sensitivity calculated when CNVfinder was applied to the corrected data and blue lines/points represent the sensitivity calculated when CNVmix was applied to the corrected data. The middle and lower plots show the specificity (with confidence intervals) and FDR, respectively, for the same experiments. The annotation and color scheme is the same as described above. [file gb-2007-8-10-r228-S6.pdf]

**S20**

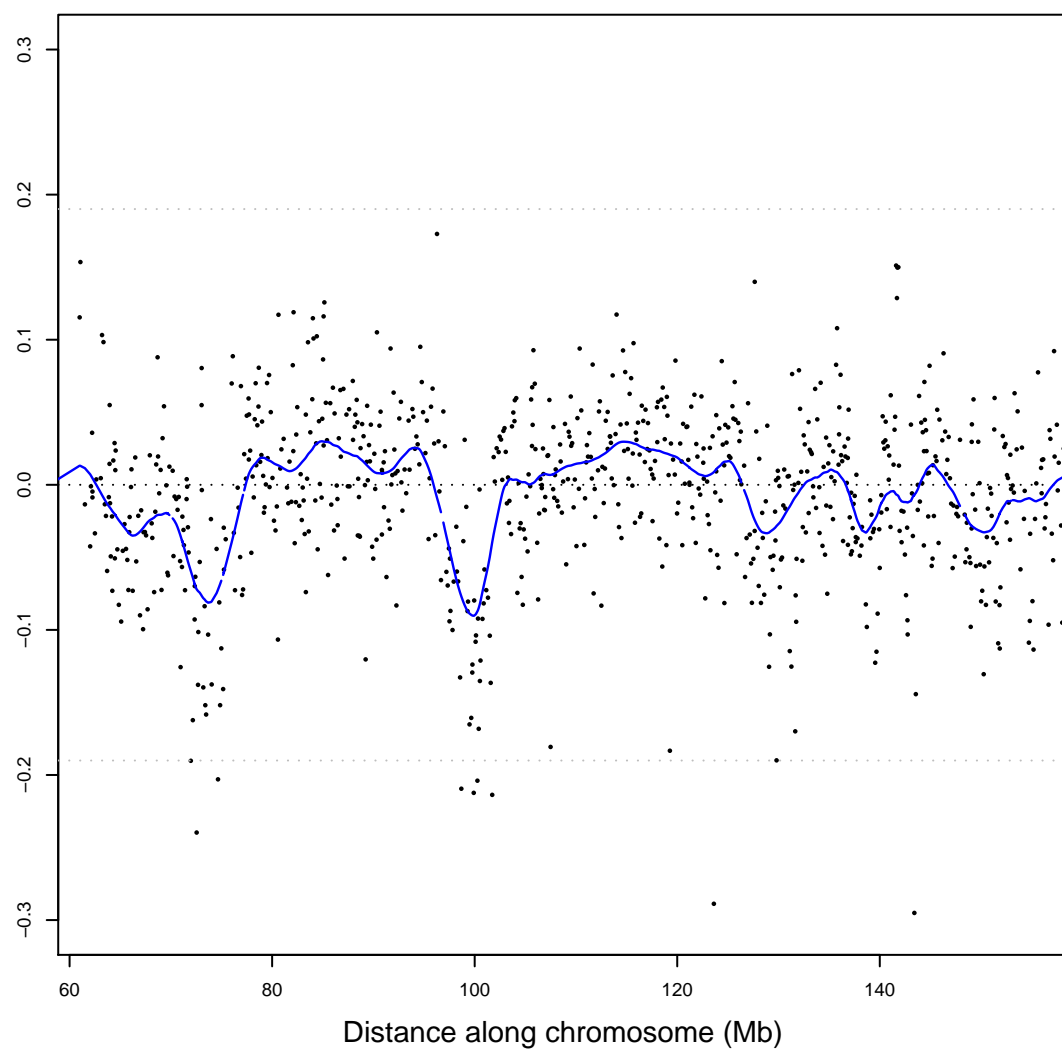

S30

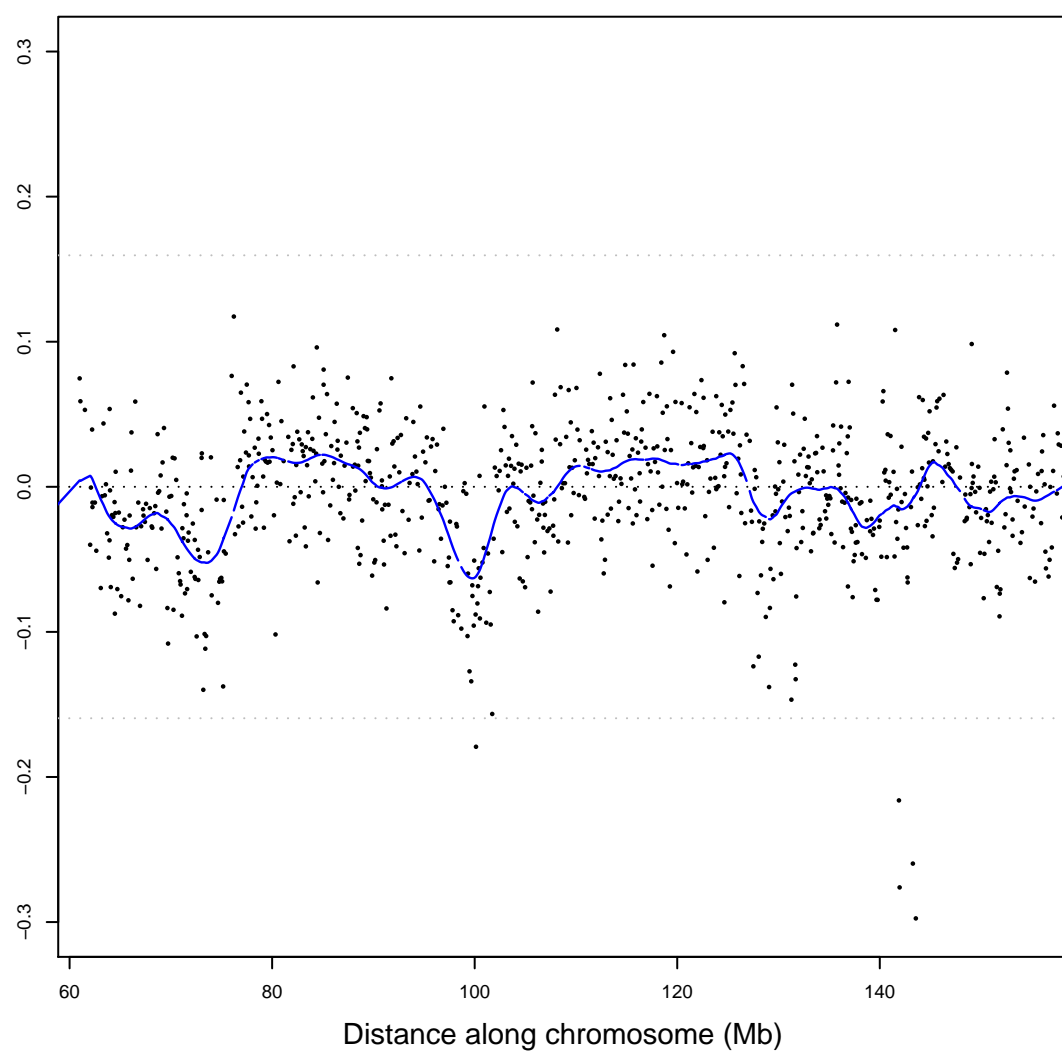

S32

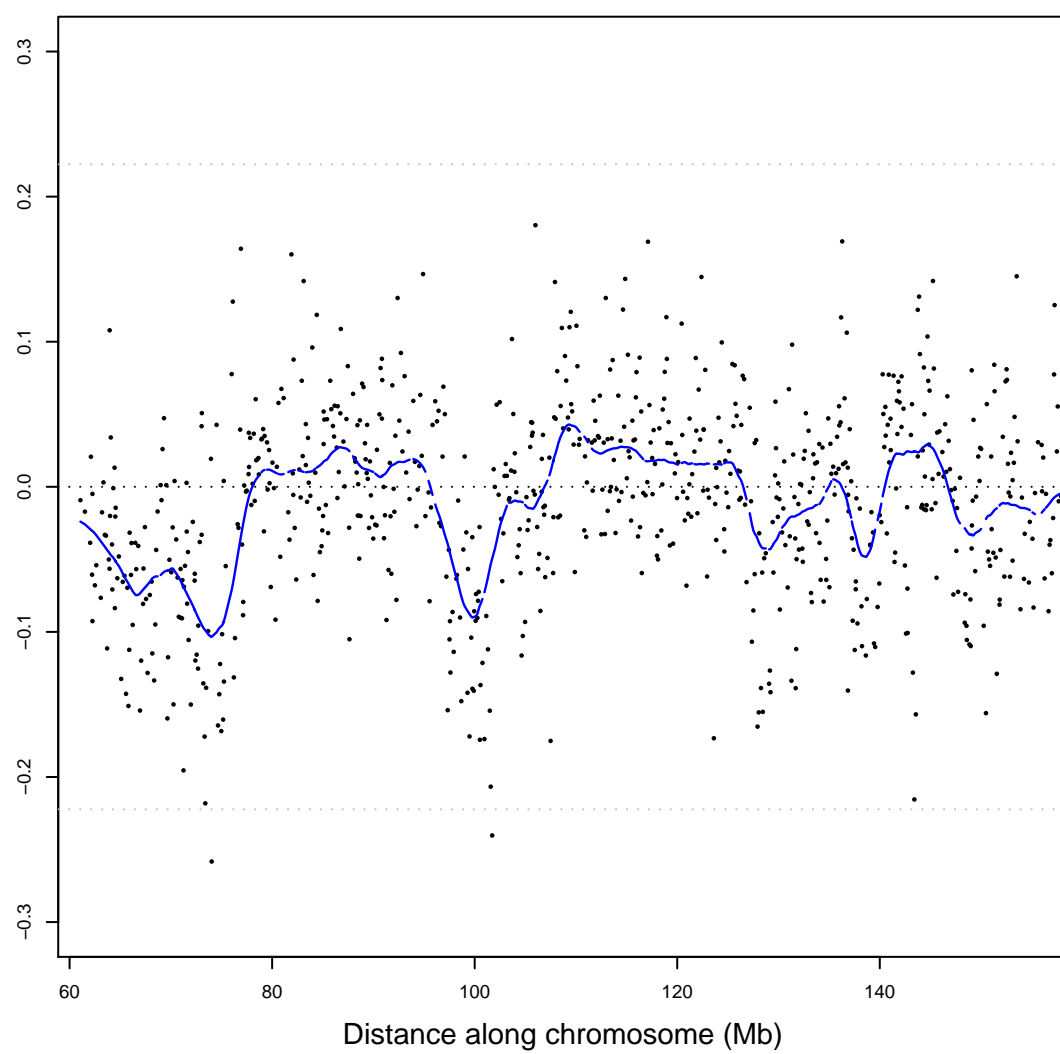

S40

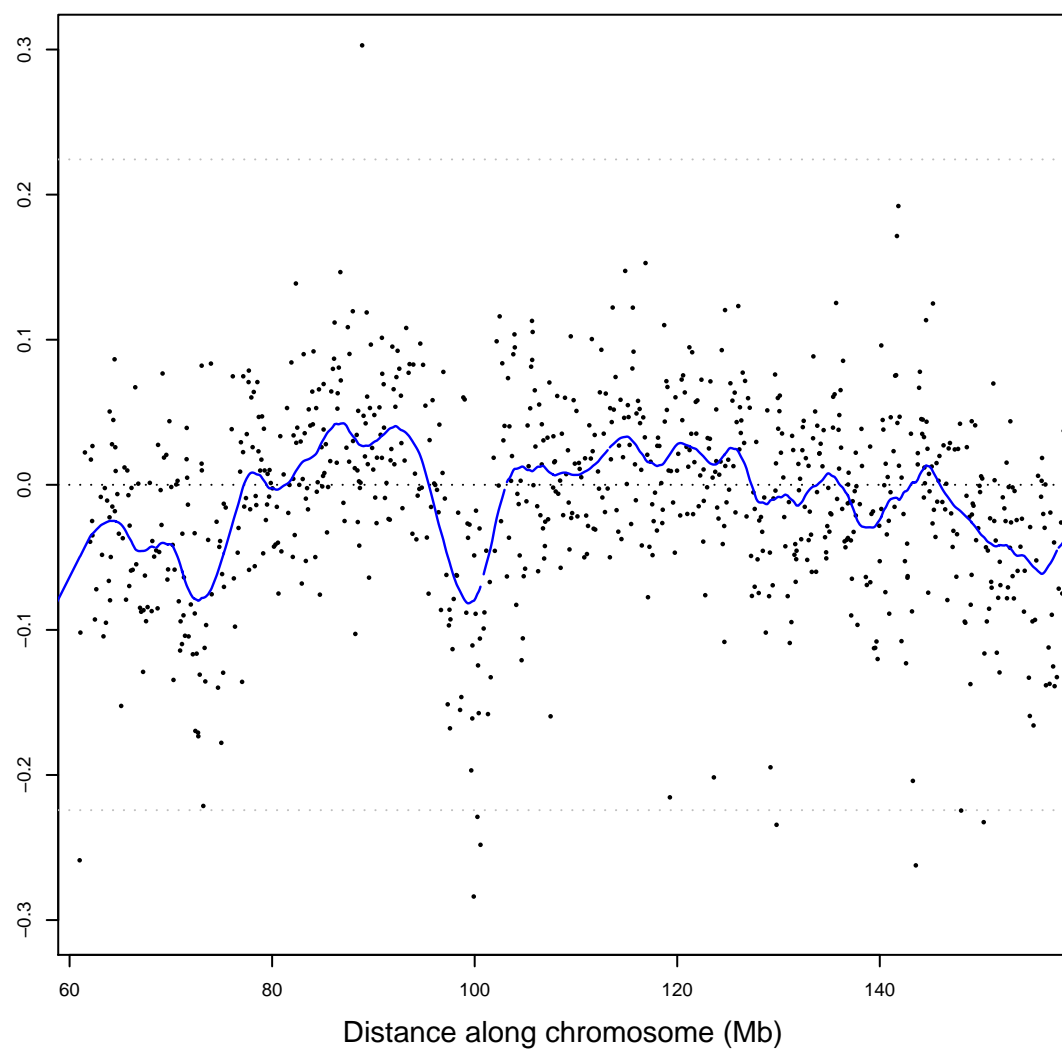

S60

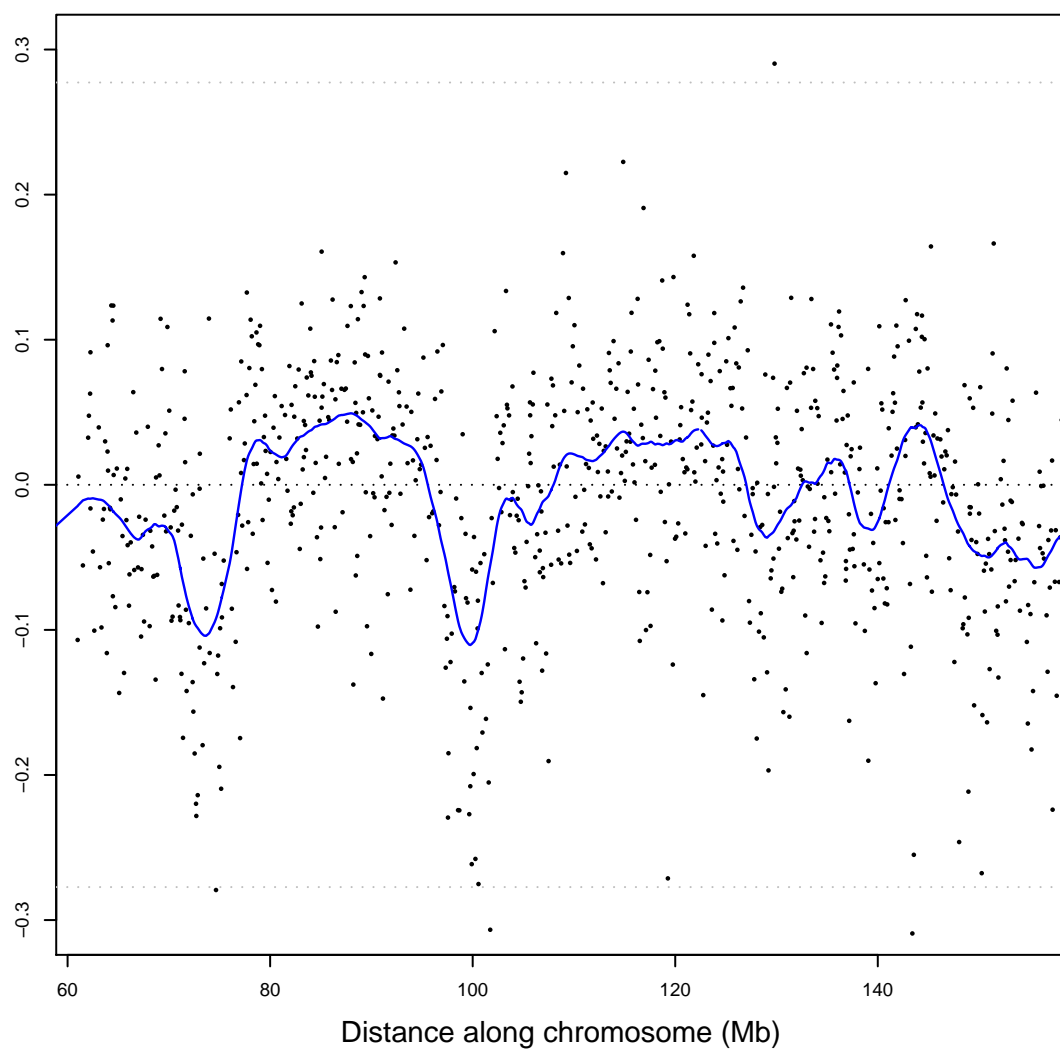

Supplement: Additional data file 10 — Each page of the PDF contains a plot of the log2 ratios for clones (censored at ±0.3) on the long arm of chromosome 7 for five samples analyzed in [15] (samples S20, S30, S32, S40 and S60). The fitted loess curve for this genomic region has been overlaid in blue and the thresholds used in [15] to identify clones harboring CNVs are shown by horizontal dashed gray lines. On all five plots we can observe that the fitted loess curve has a trough at around 75 and 100 Mb (this is common to all samples - see Additional data file 6), suggesting that this is a technical artifact. Moreover, in all five plots a small number of clones in these regions have log2 ratios that are lower than the threshold and, consequently, they are flagged (almost certainly incorrectly) as harboring a CNV. [file gb-2007-8-10-r228-S10.pdf]
