# Supplementary material for: Breaking the waves: improved detection of copy number variation from microarray-based comparative genomic hybridization
Source: Genome Biol. 2007 Oct 25;8(10):R228. doi: 10.1186/gb-2007-8-10-r228 (PMC2246302; doi:10.1186/gb-2007-8-10-r228)
Supplement: Additional data file 5 — The columns of the table give the number of clones on chromosome 4 with log2 ratios outside a threshold of ±0.06 for the three samples (NA11829, NA12044 and NA19093) shown in Figure 5. The rows indicate the number of clones that are identified using this threshold for uncorrected and corrected log2 ratios. A threshold of ±0.06 is necessary in order to identify the red clone that represents a genuine CNV in Figure 5 (log2 ratios of 0.071, -0.0665, 0.0835 prior to wave correction, 0.064, -0.069, 0.085 after wave correction). [file gb-2007-8-10-r228-S5.pdf]

Number of outlier clones identified using the threshold			
	NA11829	NA12044	NA19093
Before Wave Correction	106	121	122
After Wave Correction	79	68	80
