# Supplementary material for: Breaking the waves: improved detection of copy number variation from microarray-based comparative genomic hybridization
Source: Genome Biol. 2007 Oct 25;8(10):R228. doi: 10.1186/gb-2007-8-10-r228 (PMC2246302; doi:10.1186/gb-2007-8-10-r228)

Chromosome 1

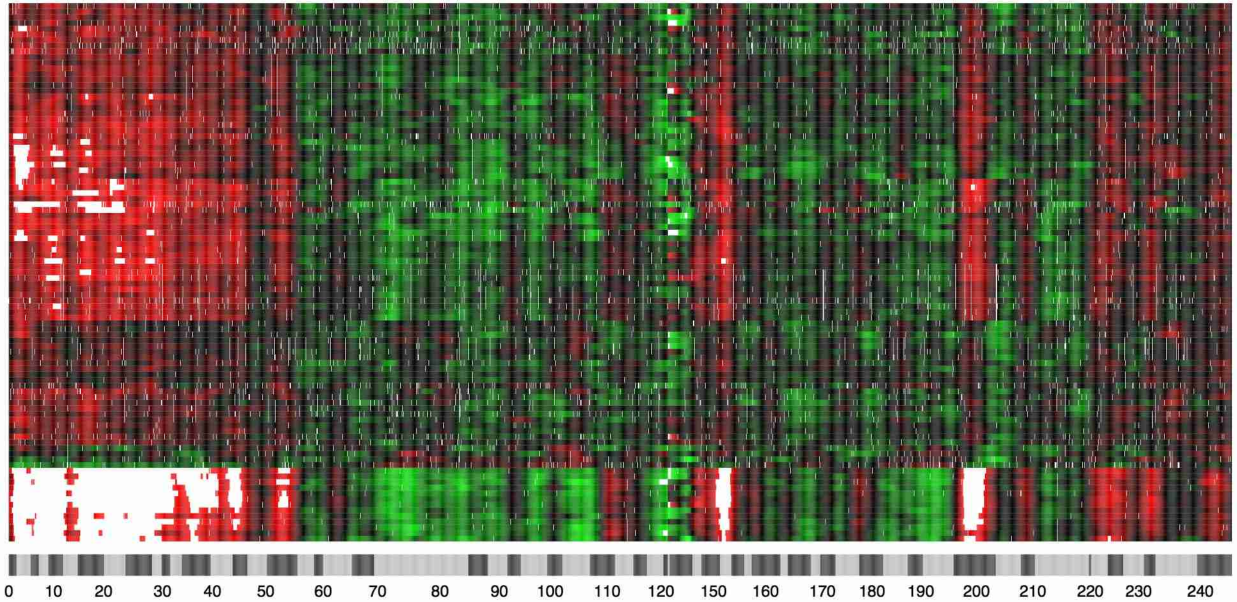

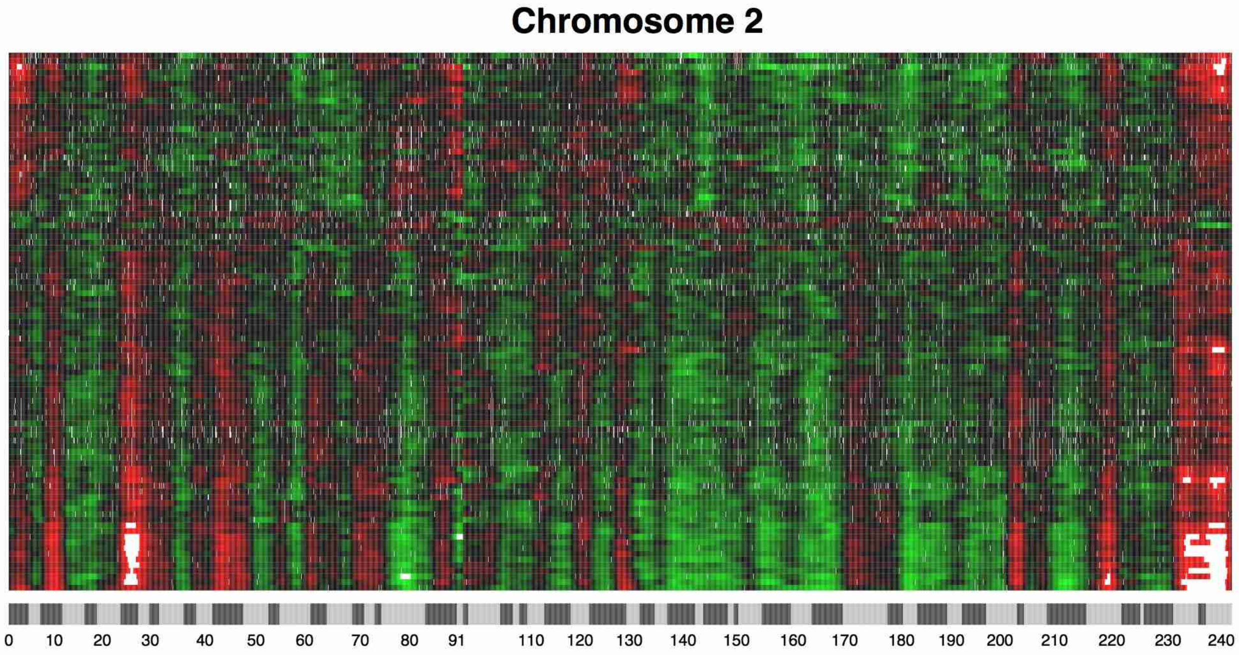
Chromosome 2
0
10
20
30
40
50
60
70
80
91
110
120
130
140
150
160
170
180
190
200
210
220
230
240

Chromosome 3

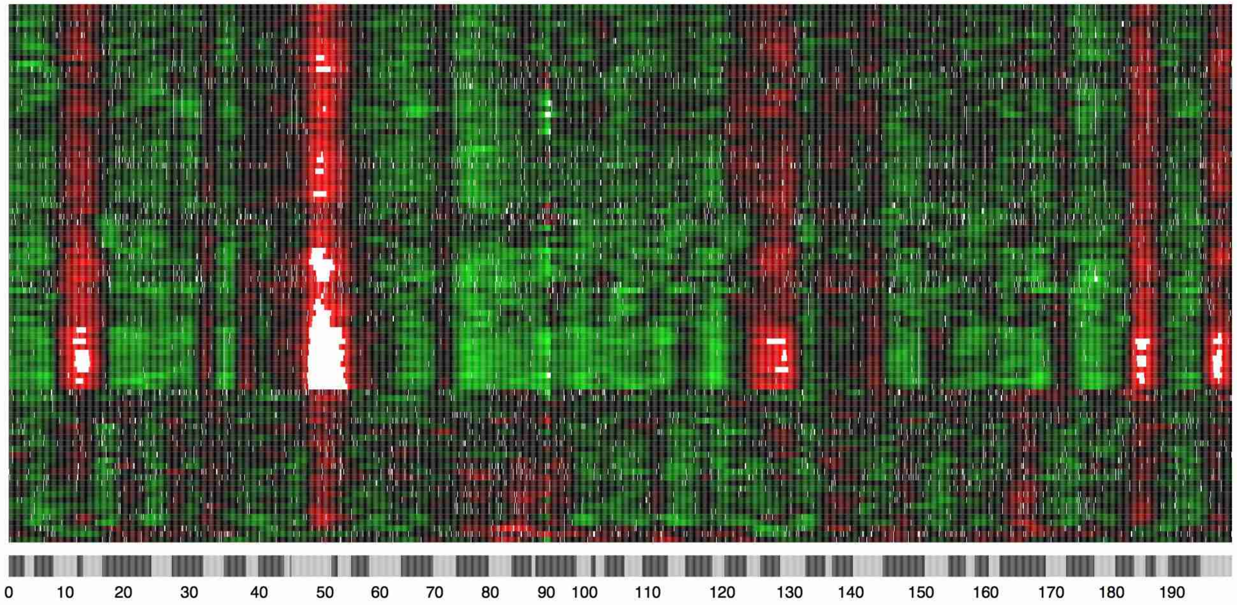

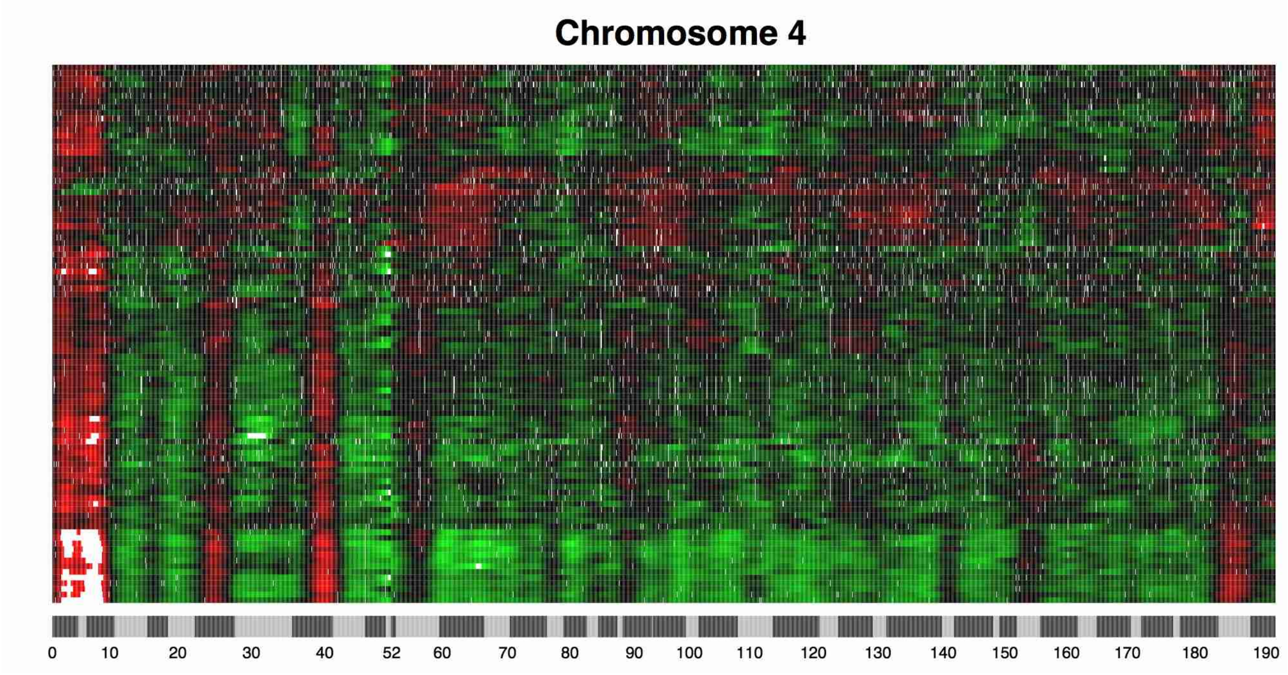
Chromosome 4
0
10
20
30
40
52
60
70
80
90
100
110
120
130
140
150
160
170
180
190

Chromosome 5

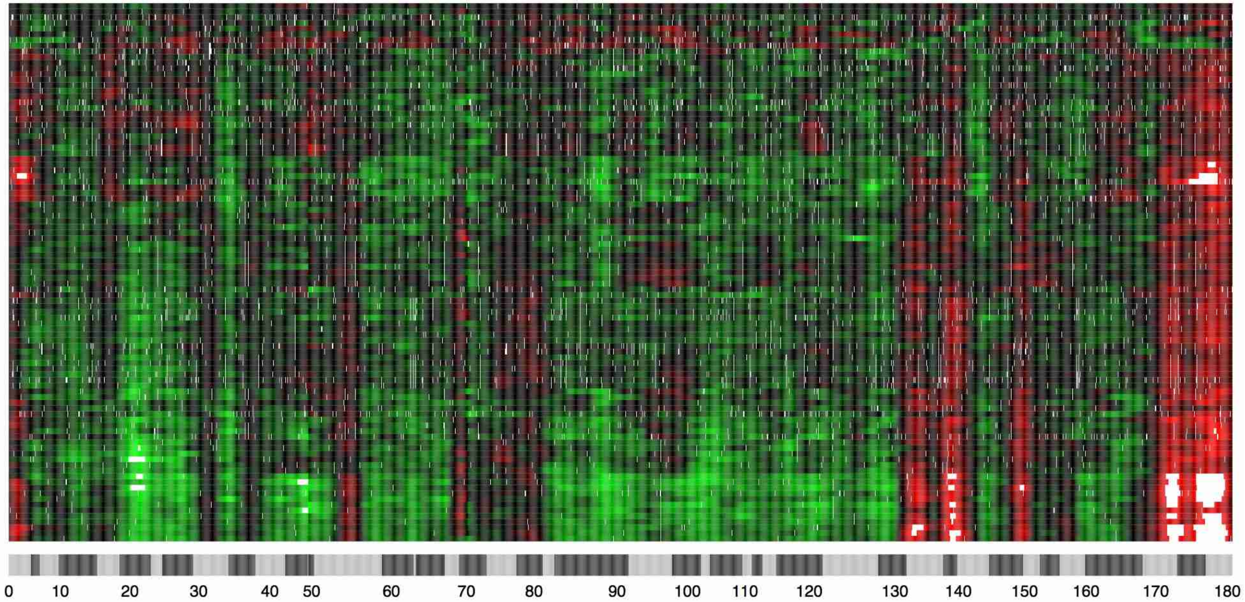

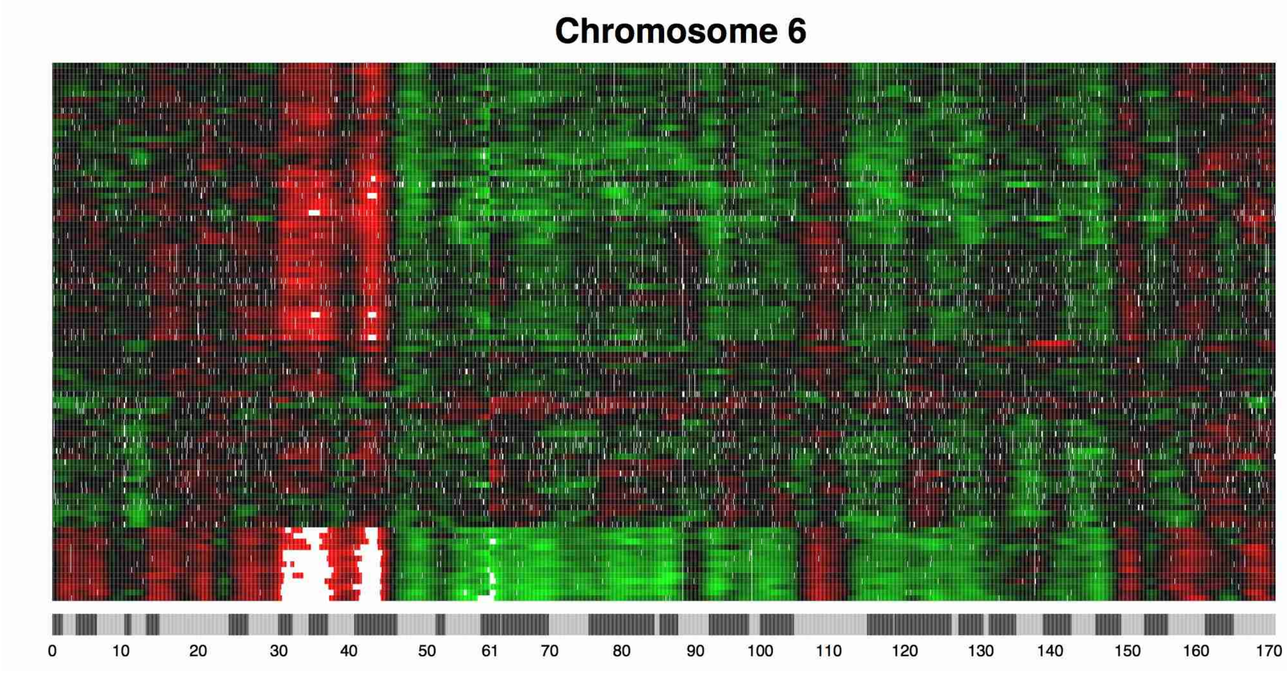
Chromosome 6
0
10
20
30
40
50
61
70
80
90
100
110
120
130
140
150
160
170

Chromosome 7

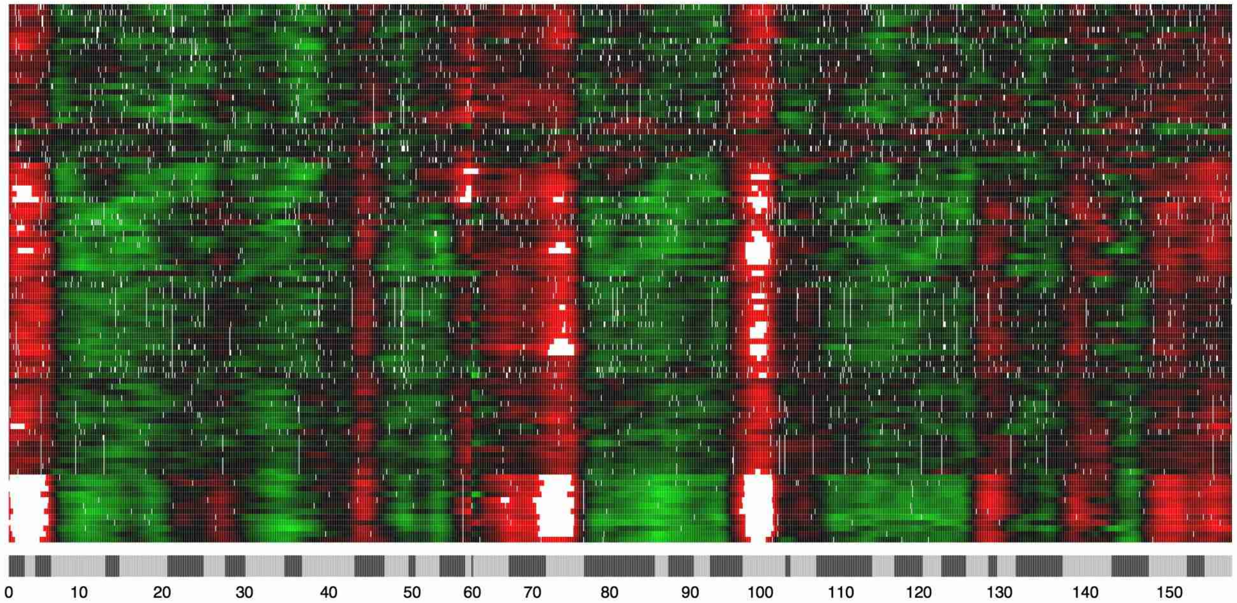

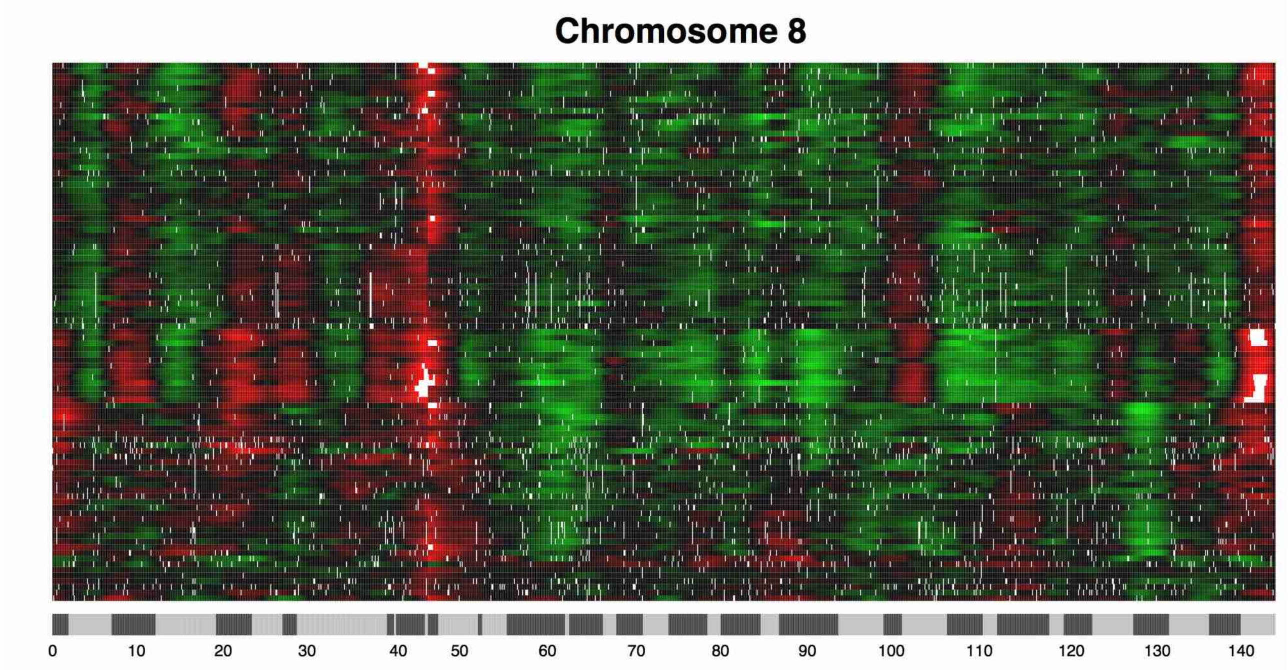
Chromosome 8
0
10
20
30
40
50
60
70
80
90
100
110
120
130
140

Chromosome 9

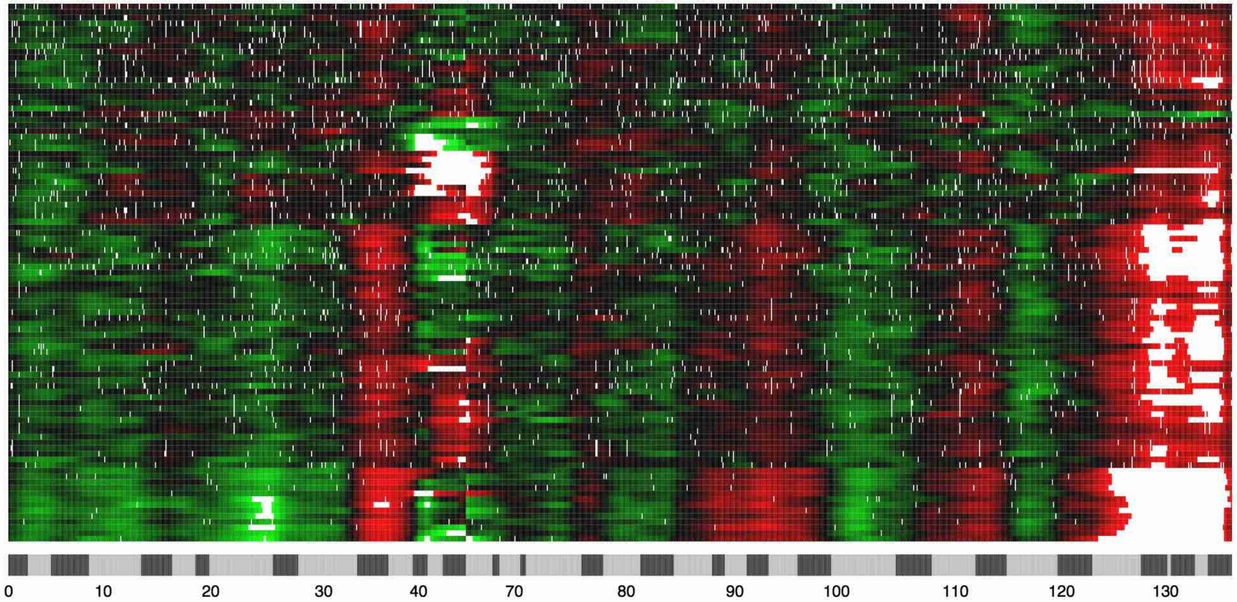

Chromosome 10

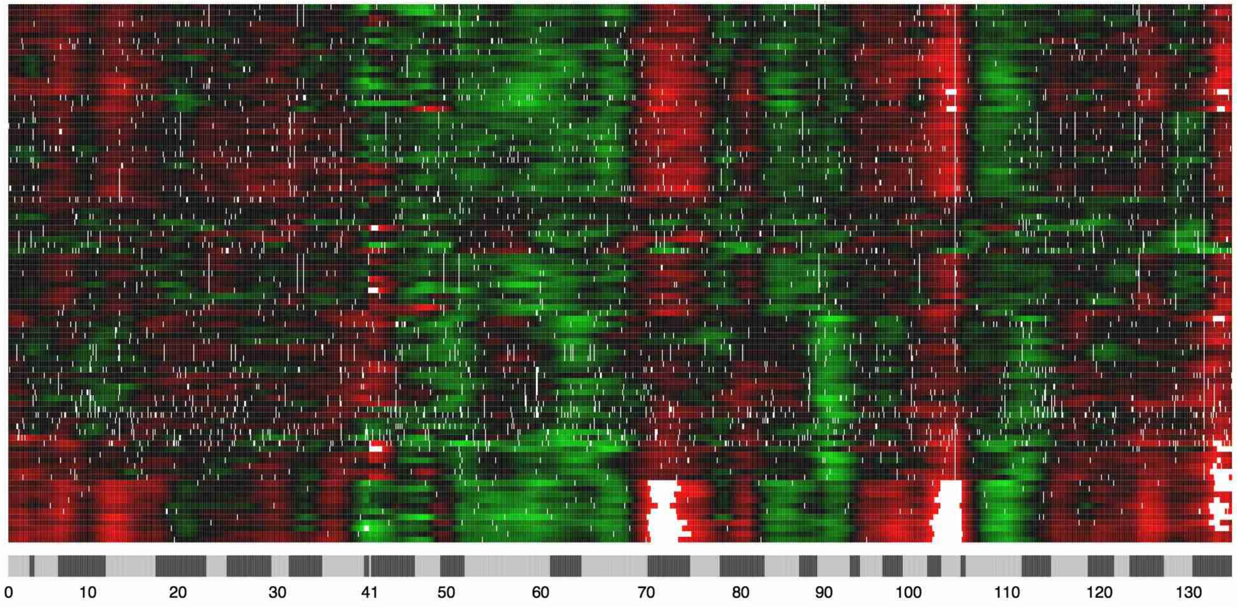

Chromosome 11

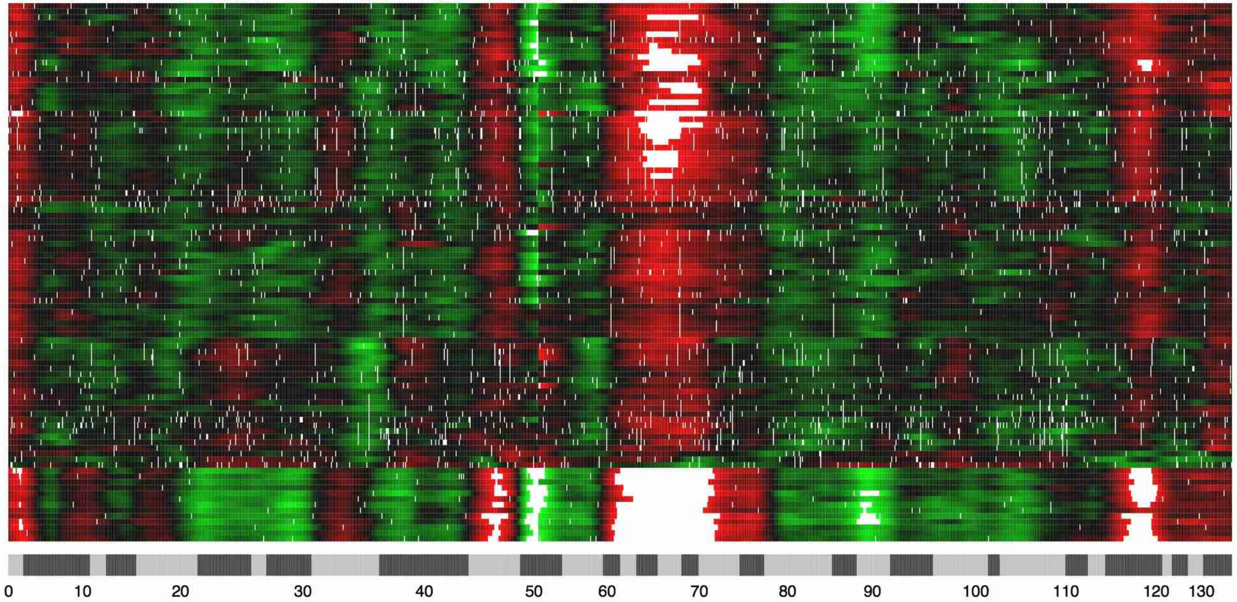

Chromosome 12

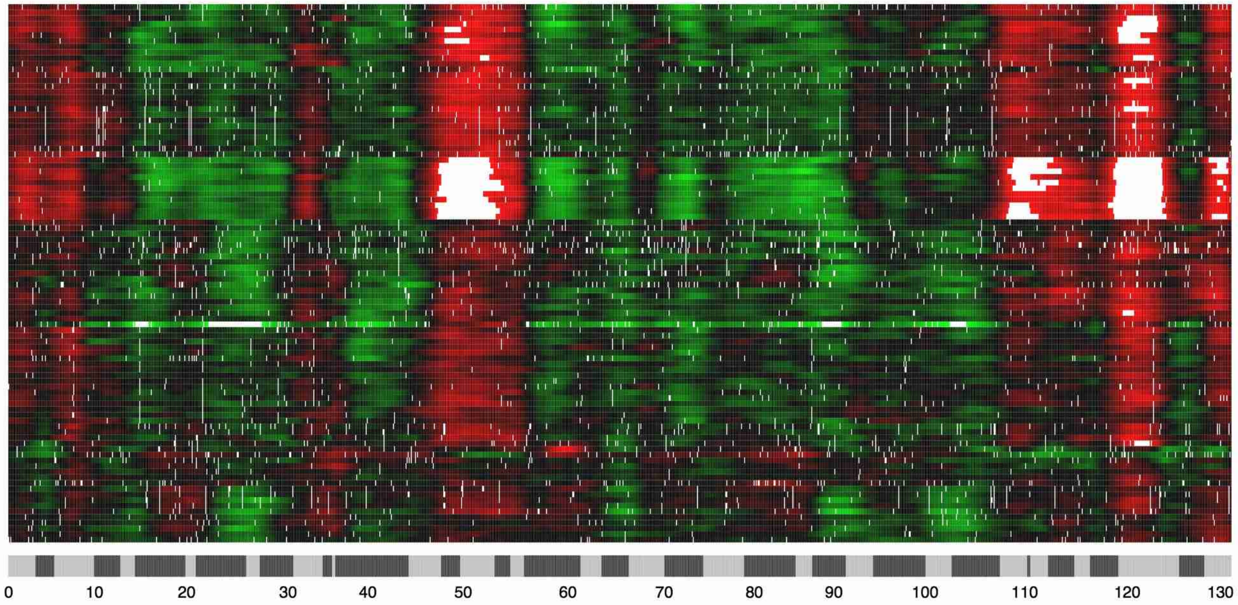

Chromosome 13

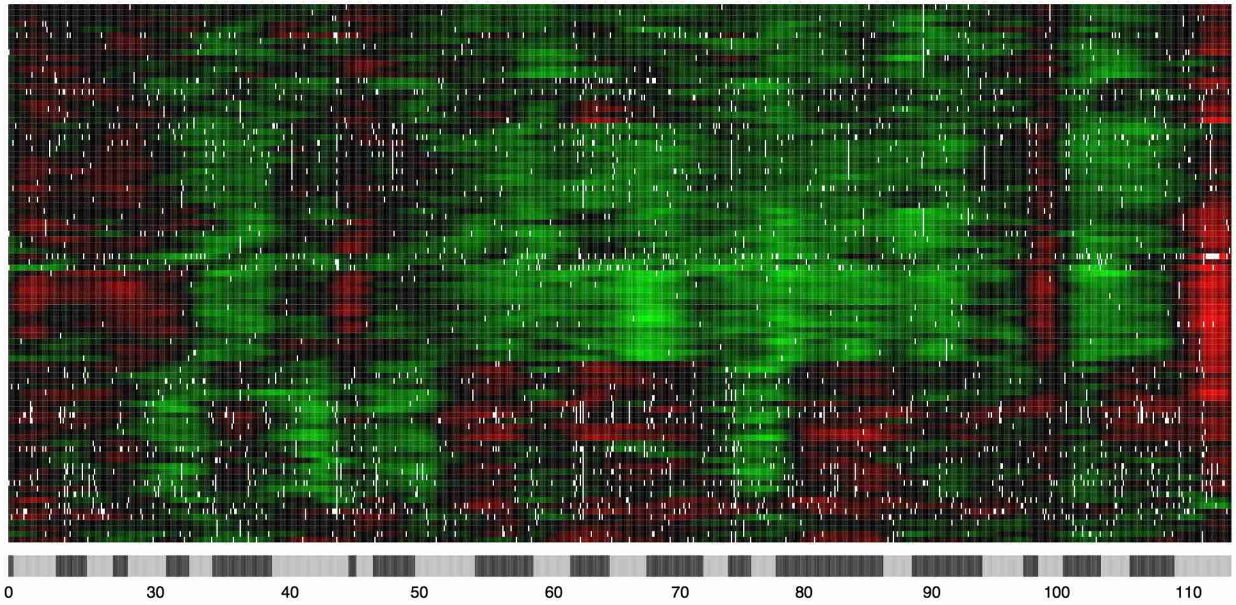

Chromosome 14

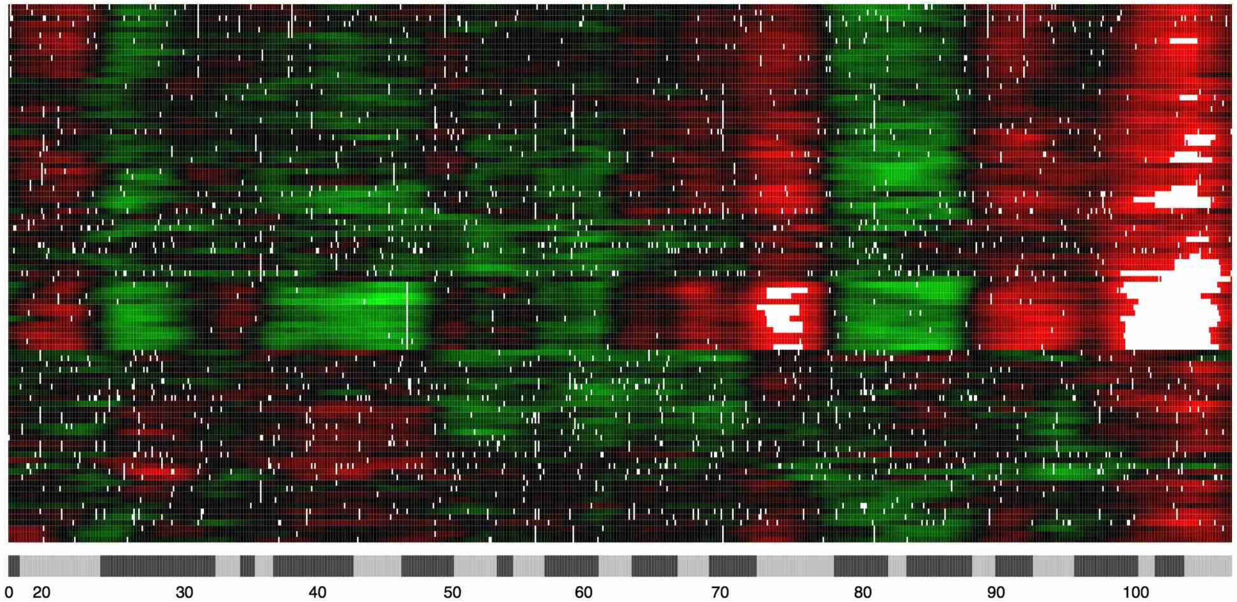

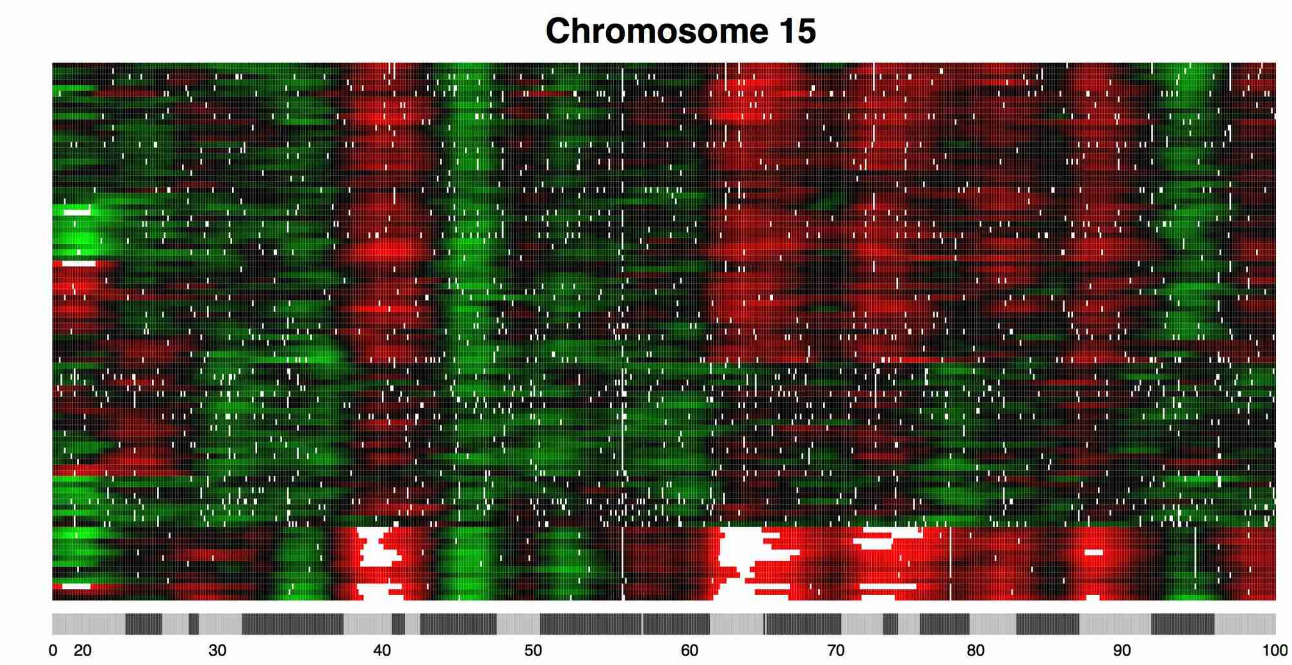

Chromosome 16

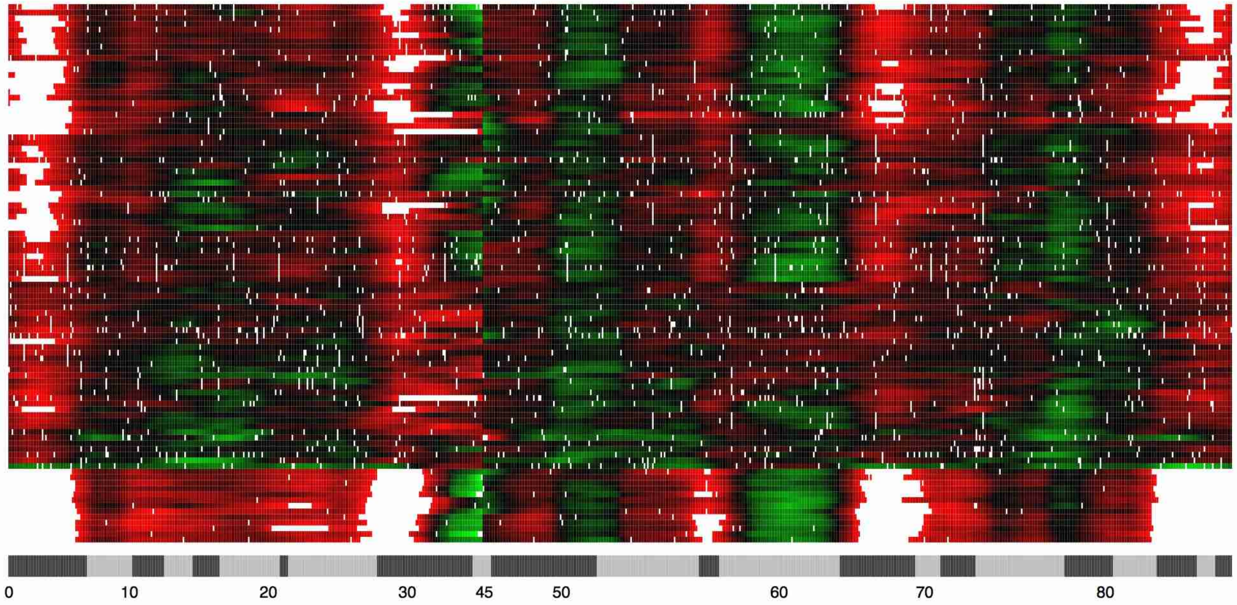

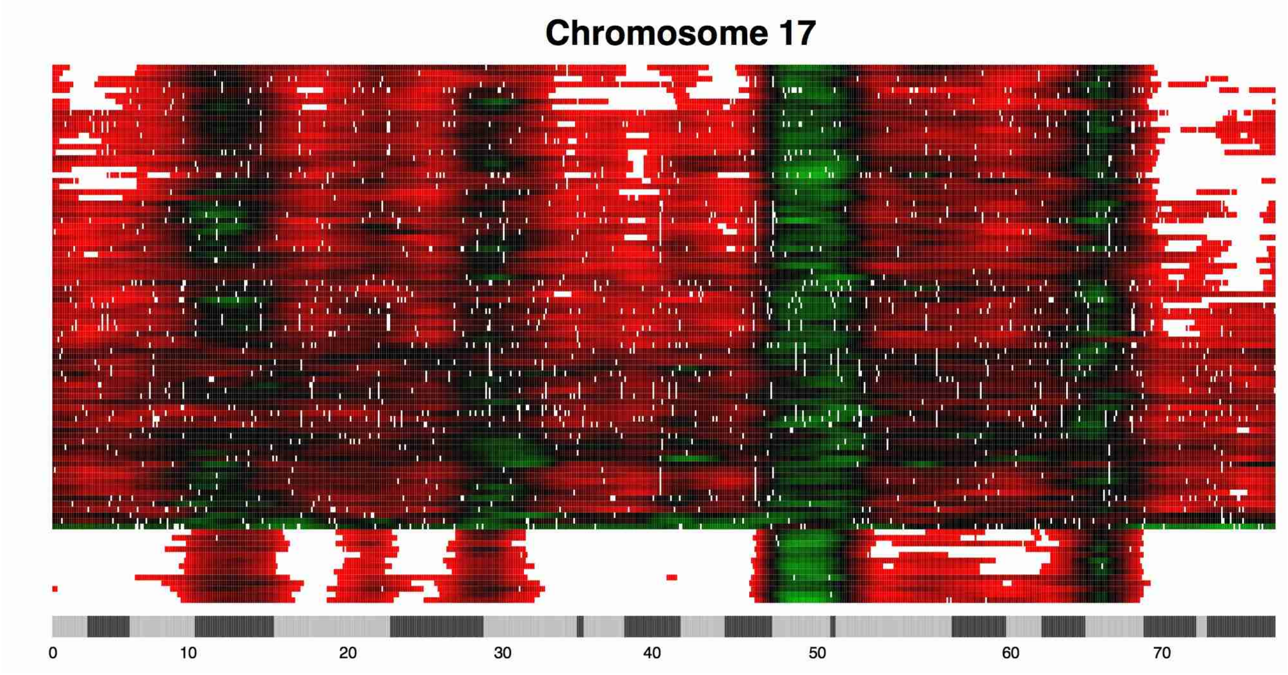
Chromosome 17
0
10
20
30
40
50
60
70

Chromosome 18

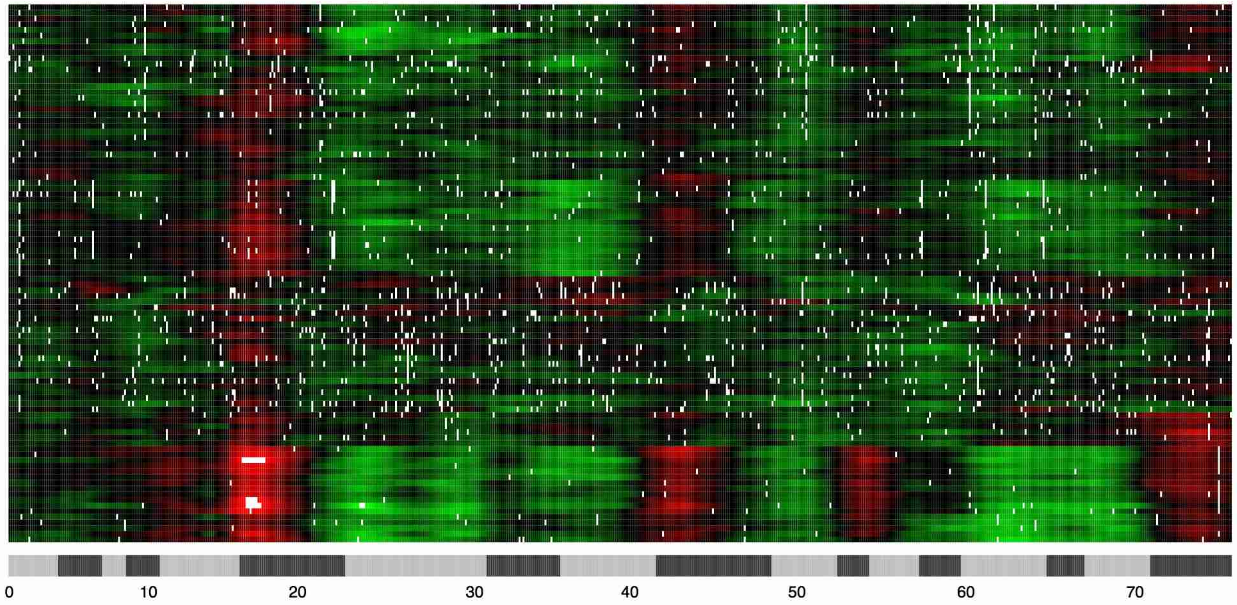

Chromosome 19

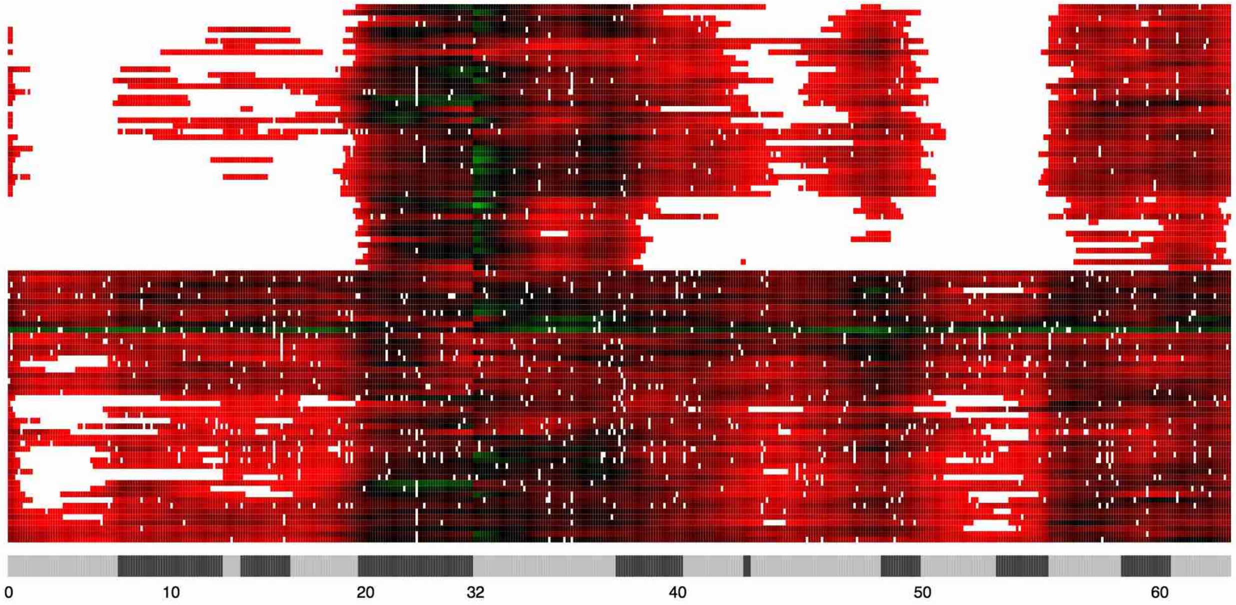

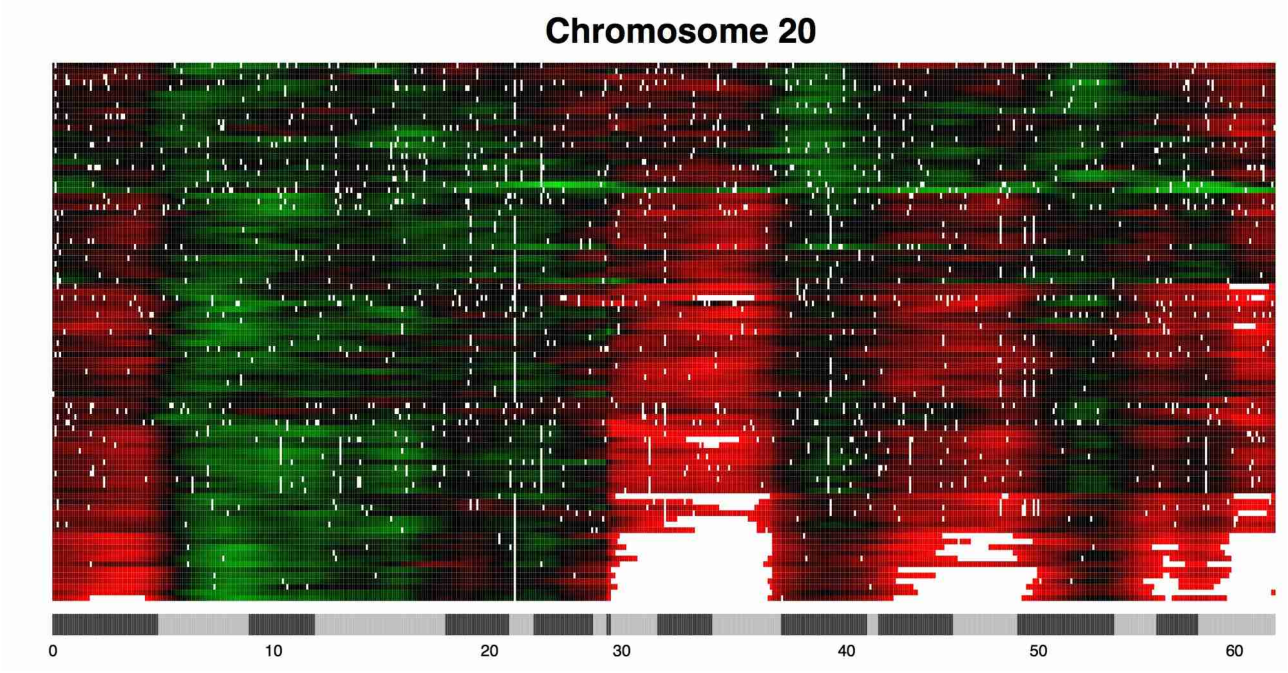
Chromosome 20
0
10
20
30
40
50
60

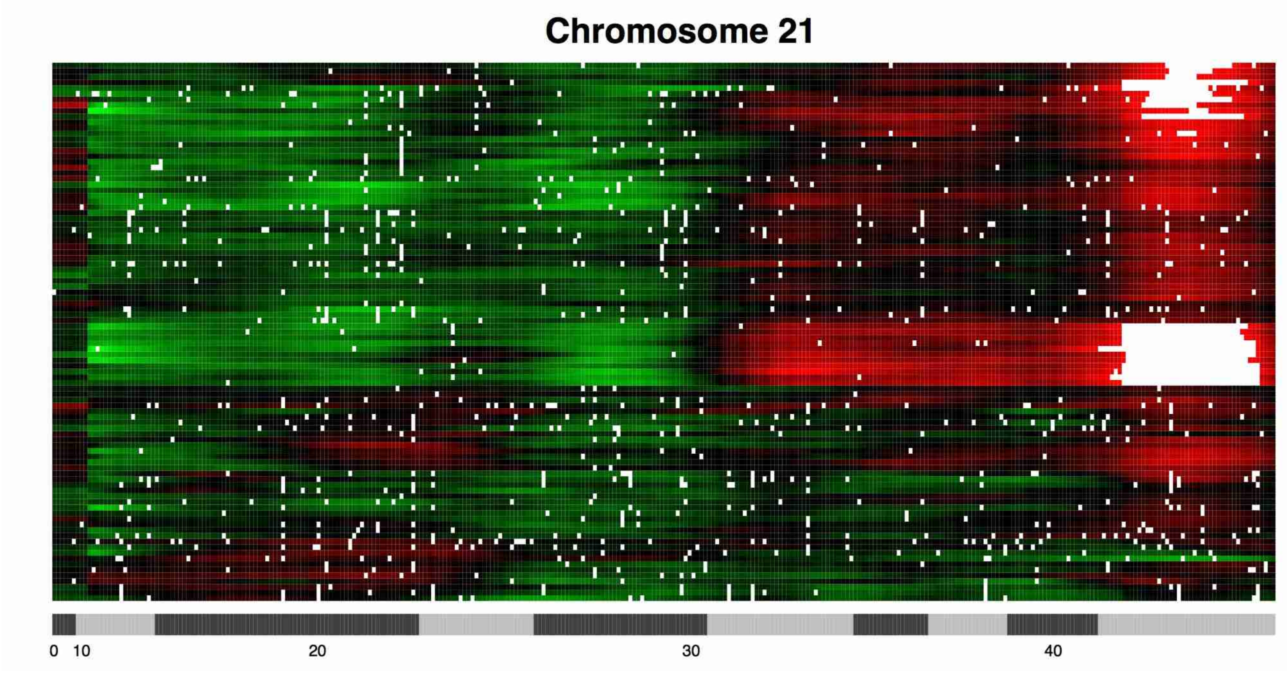
Chromosome 21
0
10
20
30
40

Chromosome 22

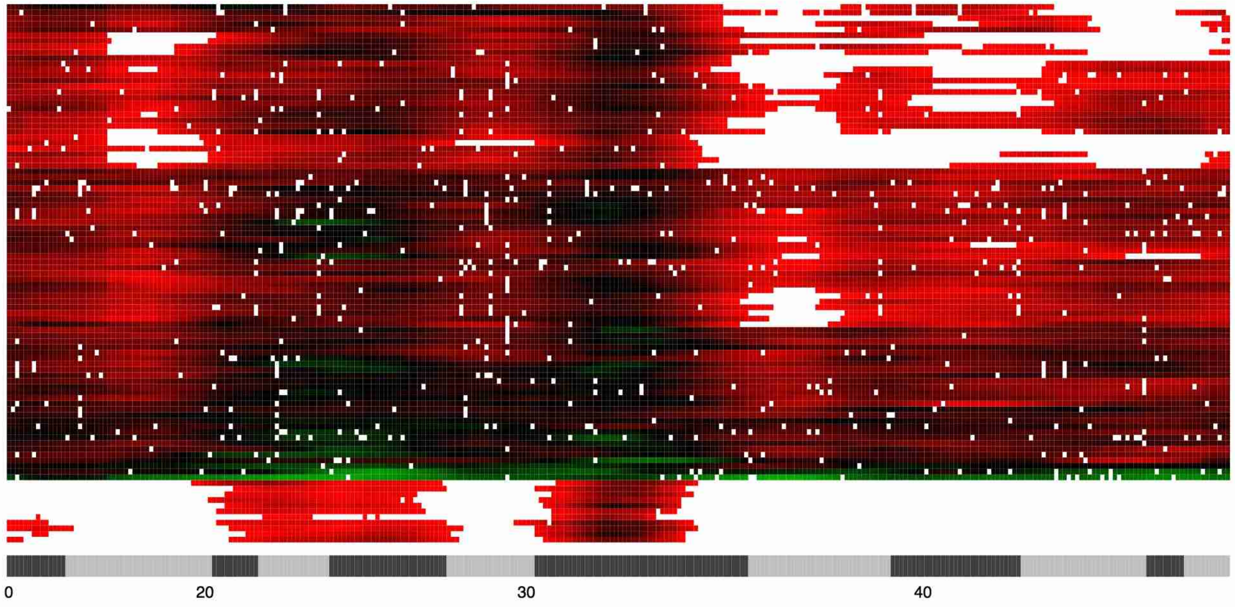

Supplement: Additional data file 9 — Each page of the PDF file corresponds to an individual chromosome. On each page the clones on a chromosome are ordered along the x-axis and the 95 samples that were investigated for CNV in [15] are plotted on the y-axis. (Note that we could not obtain mapping information for 1% of the clones and so they were removed from our analysis.) A green/red region on the heatmap indicates that the fitted loess values in this region are consistently greater/less than zero. The samples have been ordered using the Ward agglomeration method and a Euclidean distance metric. The scale along the bottom of each figure gives the location of the cytobands on a chromosome. Note that some of the heatmaps are predominantly red (noticeably chromosomes 19 and 22) - this is because the median log2 ratio is consistently less than 0 for these chromosomes. [file gb-2007-8-10-r228-S9.pdf]
