# Supplementary material for: Breaking the waves: improved detection of copy number variation from microarray-based comparative genomic hybridization
Source: Genome Biol. 2007 Oct 25;8(10):R228. doi: 10.1186/gb-2007-8-10-r228 (PMC2246302; doi:10.1186/gb-2007-8-10-r228)
Supplement: Additional data file 11 — Details of the CNVmix mixture model. [file gb-2007-8-10-r228-S11.doc]

A one-dimensional mixture model

The one-dimensional mixture model utilised by CNVmix can be formulated as follows. Assume that the data (i.e. the log_2_ ratios for all 269 hybridisation for a particular clone) is contained in an n-dimensional vector, . We then assume that has a distribution $f\left( x|m,s,p \right)$ such that

where is a Normal distribution with mean and variance , is the probability that an observation belongs to the component and is the number of components. Each component has a separate mean, but the variance can be fixed for all components.

The model/number of components fitted to each vector is determined using the Bayesian Information Criterion or BIC. The BIC is defined as

where is the maximized log likelihood for the data and the model $\left( M \right)$, is the number of parameters for model $M$, and is the length of . The chosen model is the one with the largest BIC. Optimizing the likelihood is straightforward and can be done using an EM algorithm. After finding the optimal model, each entry of $x$ is assigned to the component for which its conditional probability of belonging is highest. In practice, we fitted this model using the R library *mclust* [25,26,27,28,29,30,31,32] — our code for fitting the model is publicly available.
